# The glucocorticoid receptor associates with the cohesin loader NIPBL to promote long-range gene regulation

**DOI:** 10.1126/sciadv.abj8360

**Published:** 2022-03-30

**Authors:** Lorenzo Rinaldi, Gregory Fettweis, Sohyoung Kim, David A. Garcia, Saori Fujiwara, Thomas A. Johnson, Theophilus T. Tettey, Laurent Ozbun, Gianluca Pegoraro, Michele Puglia, Blagoy Blagoev, Arpita Upadhyaya, Diana A. Stavreva, Gordon L. Hager

**Affiliations:** 1 Laboratory of Receptor Biology and Gene Expression, National Cancer Institute, National Institutes of Health, Bethesda, MD 20892, USA.; 2 Department of Physics, University of Maryland, College Park, MD 20742, USA.; 3 High-Throughput Imaging Facility (HiTIF), Center for Cancer Research (CCR), NCI/NIH, Bethesda, MD 20892, USA.; 4 Department of Biochemistry and Molecular Biology, University of Southern Denmark, Odense, Denmark.; 5 Institute for Physical Science and Technology, University of Maryland, College Park, MD 20742, USA.

## Abstract

The cohesin complex is central to chromatin looping, but mechanisms by which these long-range chromatin interactions are formed and persist remain unclear. We demonstrate that interactions between a transcription factor (TF) and the cohesin loader NIPBL regulate enhancer-dependent gene activity. Using mass spectrometry, genome mapping, and single-molecule tracking methods, we demonstrate that the glucocorticoid (GC) receptor (GR) interacts with NIPBL and the cohesin complex at the chromatin level, promoting loop extrusion and long-range gene regulation. Real-time single-molecule experiments show that loss of cohesin markedly diminishes the concentration of TF molecules at specific nuclear confinement sites, increasing TF local concentration and promoting gene regulation. Last, patient-derived acute myeloid leukemia cells harboring cohesin mutations exhibit a reduced response to GCs, suggesting that the GR-NIPBL-cohesin interaction is defective in these patients, resulting in poor response to GC treatment.

## Teaser

Transcription factors associate with architectural proteins to regulate genome organization and three-dimensional gene regulation.

## INTRODUCTION

Dynamic remodeling of nuclear architecture is paramount during development, stem cell differentiation, and environmental adaptation (*1*, *2*). The cohesin complex is a master regulator of genome organization, regulating sister chromatid cohesion, DNA repair, and nuclear topology. Besides its prolonged binding at TAD boundaries (*3*–*7*), cohesin is also present at enhancer-promoter contacts (*2*, *6*–*9*). Enhancer-promoter interactions are dynamically controlled by lineage-specific transcription factors (TFs) that ensure proper phenotypic gene expression. This suggests a possible regulatory connection between TFs and the architectural cohesin complex (*2*). In mammals, the cohesin complex is formed by protein subunits SMC1a (structural maintenance of chromosomes 1a), SMC3, and RAD21. In addition, a variety of accessory proteins such as NIPBL, Mau2, SA1/SA2, Wapl, and PDS5 regulate cohesin genome functions. The main cohesin subunits SMC1a, SMC3, and RAD21 bind stoichiometrically at chromatin at a 1:1:1 ratio to form the ring-shaped complex. However, a large fraction (60 to 80%) of these subunits are not associated with chromatin, suggesting that cohesin loading is a dynamic process (*5*, *10*–*12*). SCC2/NIPBL dynamically loads cohesin to chromatin accessible sites (*13*), guiding the bidirectional loop extrusion mechanism toward CTCF anchors in an adenosine 5′-triphosphate (ATP)–dependent manner (*2*, *4*, *13*–*15*). The genome localization and nuclear topology functions of the NIPBL-cohesin complex are modulated by transcriptional processes (*16*, *17*). However, the mechanism behind the interplay between transcriptional machinery and the cohesin complex is still not well understood. Recent biophysical studies revealed that nucleosomes and other protein complexes restrict cohesin translocation (*18*), suggesting that transcriptional processes and chromatin remodelers could promote the cohesin translocation along the DNA strand (*18*, *19*).

Cohesin complex function and transcriptional processes are in some ways correlated, but acute depletion of the cohesin complex does not alter the global RNA production in mammalian cells (*6*). Considering that most of these studies are performed under steady-state conditions, they only address the impact of cohesin depletion on the basal transcriptional activity (*6*, *20*). The cohesin complex has been shown to bind at chromatin accessible sites bound by the estrogen receptor (ER) (*8*, *21*) and to modulate ER-mediated transcriptional activity (*8*). Furthermore, the activation of steroid hormone receptors results in changes in hormone-dependent chromatin looping (*21*, *22*), suggesting a profound relationship between steroid hormone receptor actions and genome organization. However, how cohesin regulates gene expression in response to induction is still under discussion (*23*).

Here, we investigate the interplay between the cohesin complex and TFs, using the glucocorticoid (GC) receptor (GR), as model system. GR is a nuclear receptor known to translocate from the cytoplasm to the nucleus upon ligand treatment. Previous studies have shown that treatment with GCs strengthens chromatin interactions; these could be either stable or dynamic (*21*, *22*, *24*). GR binds preestablished chromatin loops enriched for cohesin subunits Rad21 and Smc3 but depleted by CTCF, suggesting a possible connection between cohesin and GR activity (*22*). Here, we show that chromatin-bound GR interacts with the cohesin loader NIPBL and demonstrate that GC treatment induces NIPBL recruitment to enhancers, promoting cohesin complex chromatin binding.

Although most of the current work uses GR as a model TF, we suggest that this mechanism may be applicable to many other transcriptional regulators capable of interacting with NIPBL. Together, our data reveal a general mechanism by which TFs regulate the three-dimensional organization of the genome.

## RESULTS

### Loop extrusion is triggered in a hormone-dependent manner

**Fig. 1.**
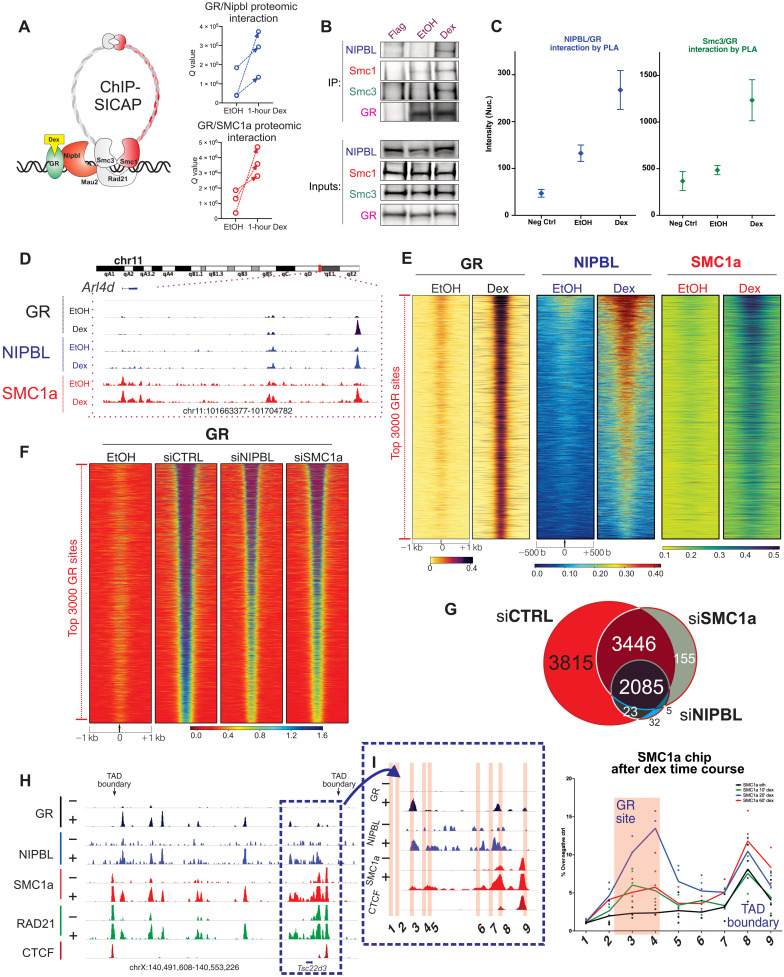
NIPBL and cohesin are loaded onto chromatin in a hormone-dependent manner. (**A**) GR-cohesin–interacting partners identified by GR ChIP-SICAP. GR interacts significantly with NIPBL and SMC1a, marked in red. (**B**) GR-immunoprecipitation (and FLAG-IP) and blotting against SMC1, SMC3, NIPBL, and GR after 1 hour of EtOH or dexamethasone treatment in mouse breast adenocarcinoma cells. (**C**) Endogenous interaction, measured by proximity ligation assay, between GR and NIPBL (left) and SMC3 (right). (**D**) GR, NIPBL, and SMC1a ChIP-seq after 1 hour of EtOH or dexamethasone treatment. (**E**) Heatmaps representing GR, NIPBL, and SMC1a ChIP-seq at the 3000 strongest GR locations previously identified in 3134 cells before and after 1 hour of 100 nM dexamethasone. Data combine two biological replicates normalized to 10 million reads. (**F**) Heatmap of GR ChIP-seq intensity at the 3000 strongest GR locations [same as (E)] in siCTRL, siSMC1a, and siNIPBL cells. Genomic data are normalized [fragments per kilobase million (fpkm)] to a total of 10 million reads and further to local tag density (*P* < 0.000001 from Wilcoxon test). (**G**) Venn diagram of GR ChIP-seq peaks identified in two independent replicates of siCTRL, siSMC1a, and siNIPBL cells. (**H**) Genome browser screenshot of the Tsc22d3 gene locus for the ChIP-seq of GR, NIPBL, SMC1A, RAD21, and CTCF. The TAD boundary at the 3′ untranslated region of TSC22D3 gene was used to evaluate cohesin binding and loop extrusion mechanism mediated by GR. (**I**) Red highlighted regions correspond to PCR products designed to evaluate the loop extrusion process. On the right side, ChIP qRT-PCR for SMC1a after EtOH treatment and 20′ and 60′ of dexamethasone (data points show three biological replicates).

To uncover novel interacting partners of GR at the chromatin level, we performed quantitative label-free mass spectrometry (MS), chromatin immunoprecipitation–selective isolation of chromatin-associated proteins (ChIP-SICAP) (*25*), using mouse mammary carcinoma cells treated or untreated with dexamethasone, a steroid hormone known to rapidly translocate the steroid receptor GR to the nucleus and promote its binding to chromatin and gene regulatory functions (fig. S1A). The cohesin loader NIPBL and the cohesin subunit SMC1a are among the top interactors with the chromatin-bound GR (Fig. 1A). Independent methods, including immunoprecipitation and proximity ligation assay, confirmed the direct interaction between endogenous GR, NIPBL, and the cohesin complex (Fig. 1, B and C).

To investigate whether the NIPBL-GR interaction increases chromatin binding after hormone activation, we performed two biological replicates of ChIP sequencing (ChIP-seq) for NIPBL and SMC1a before and after 1 hour of dexamethasone treatment. ChIP-seq analysis demonstrated an increased binding of NIPBL and SMC1a upon 1-hour dexamethasone treatment at GR-bound chromatin elements (Fig. 1, D and E, and fig. S1, B, C, H, and I). Together, these data demonstrate that the steroid hormone receptor GR interacts at the chromatin level with the cohesin loader NIPBL and the cohesin arm SMC1a, promoting cohesin chromatin binding at GR sites in a hormone-dependent manner (Fig. 1, D and E).

To assess the indirect effects of GC treatment toward the cohesin complex and potential action of GC treatment on global recruitment of the cohesin complex, we performed an independent chromatin fractionation assay, followed by Western blot. We did not observe any dexamethasone-dependent global changes of NIPBL or SMC1A at the chromatin fraction (fig. S2A), suggesting that the enhanced chromatin binding of NIPBL and SMC1A is mostly exclusive to chromatin sites bound by GR after dexamethasone treatment.

To evaluate whether this interaction was important for GR chromatin binding, we performed GR ChIP-seq upon NIPBL and SMC1a knockdown. We performed small interfering RNA (siRNA) treatment against NIPBL or SMC1A in mammary breast adenocarcinoma cells. An efficient knockdown, achieved after 72 hours of siRNA transfection, was evaluated by Western blot for both NIPBL and the core cohesin subunit SMC1a (fig. S1G). Loss of NIPBL reduced GR binding to chromatin, and both the number of GR chromatin sites and the strength of GR binding were severely compromised (Fig. 1, F and G). To further validate these findings, we performed GR ChIP-seq in SMC1a knockdown cells. In agreement with our siNIPBL results, depletion of SMC1A (72 hours of siRNA treatment) significantly reduced GR binding to chromatin (Fig. 1, F and G). In support of these results, our chromatin fraction data clearly displayed a decrease in GR accumulation in the chromatin fraction after the knockdown of NIPBL and Smc1a (fig. S2A). These data suggest a multifactorial model whereby the NIPBL-cohesin complex associates with transcriptional regulators to induce long-range gene regulation (*4*, *16*, *17*, *26*).

By determining the position of TAD anchors via CTCF motif orientation analysis (*27*), we observed that GR primarily binds in close proximity to the TAD anchors (fig. S1, D and E). To test whether GR induces cohesin stalling at the TAD boundaries, we quantified the enrichment of cohesin at TAD boundaries proximal to GR-binding sites versus those distant from GR peaks. TAD boundaries near GR peaks showed a much stronger enrichment of cohesin after dexamethasone treatment (fig. S1F). This suggests that the activation of the steroid receptor promotes NIPBL-cohesin chromatin binding and loop extrusion of the cohesin in the direction of CTCF-bound TAD boundaries. To confirm our hypothesis, we performed SMC1a ChIP quantitative polymerase chain reaction (qPCR) after 10, 20, and 60 min of GC treatment to quantify the extent of cohesin chromatin binding and loop extrusion at the TSC22D3 gene locus (Fig. 1H). We found a strong enrichment at 20 min at the GR-bound site, whereas at 60 min, SMC1a was still enhanced at the proximal TAD boundary (Fig. 1I). To confirm this pattern of cohesin loading, we have repeated the assay investigating the chromatin landscape of multiple GR target genes regulated by long-range interactions. Most of the GR-bound enhancers investigated show an acute enrichment of the cohesin subunit SMC1A after only 20 min of dexamethasone compared to 60 min (fig. S2, B to D). This enrichment at GR-bound enhancers was reduced after 60 min, while it was still highly evident at the nearby TAD boundary (fig. S2, B to D).

Together, these data indicate that GR and probably other TFs can promote cohesin binding at enhancer sites by a direct interaction with NIPBL. The cohesin complex is then extruded toward the proximal TAD boundary in an ATP-dependent manner (*13*).

### NIPBL regulates inducible long-range gene regulation

**Fig. 2.**
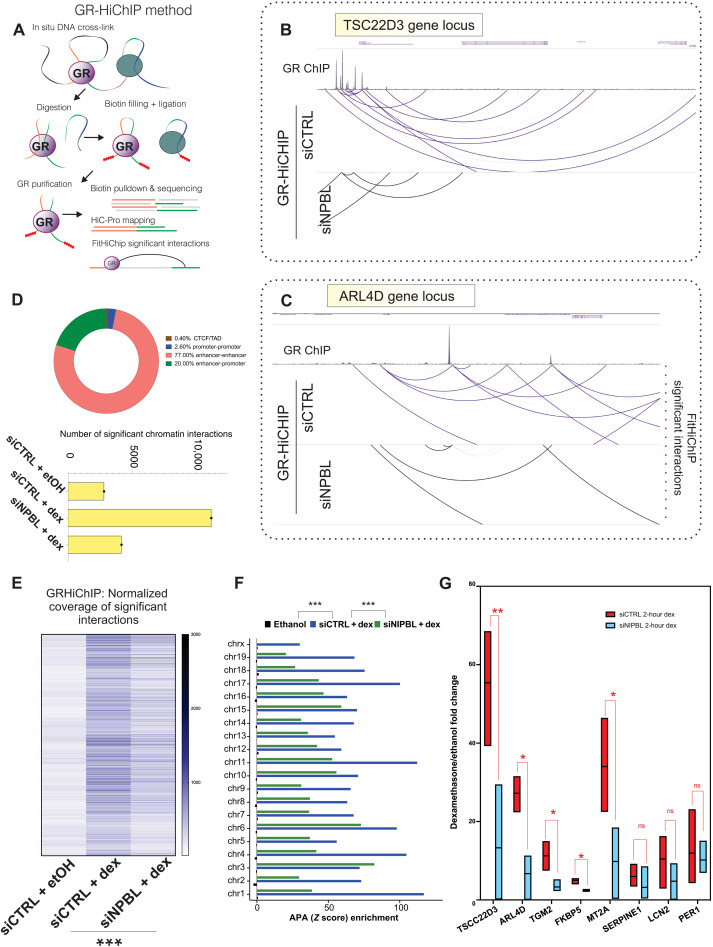
NIPBL regulates GR-bound long-range interactions and gene regulation. (**A**) Experimental design of the GR-HiChIP protocol and analysis pipeline. (**B** and **C**) WashU genome browser screenshot of GR-bound long-range interaction identified by FitHiChIP at the TSC22D3 and ARL4D locations, in siCTRL and siNIPBL cells. Loop strength is normalized at a 5-kb resolution. (**D**) Top: The number of significant GR long-range interactions identified by FitHiChIP, at a *Q* value of 0.01 using coverage bias parameters, in untreated cells, siCTRL + 1-hour dexamethasone, and siNIPBL + 1-hour dexamethasone. Bottom: The percentages of the GR-bond chromatin interactions stratified into different types of long-range interactions. (**E**) Quantification intensity of the commonly identified GR-HiChIP long-range interactions identified by FitHiChIP (*Q* value of 0.01) in siCTRL and siNIPBL samples, at a 5-kb resolution. NIPBL depletion leads to profound reduction of GR-bound chromatin interaction number and looping strength. *P* < 0.00001 derived from KS test. (**F**) Aggregate enrichment for each mouse chromosome in the GR-HiChIP datasets in EtOH, siCTRL, and siNIPBL samples. Aggregate peak analysis (APA) measurements (*Z* score) were calculated on the long-range interactions identified by FitHiChIP (*Q* value of 0.01) using the juicertools at a 5-kb resolution and VC normalization. *P* < 0.00001 derived from KS test. (**G**) Nascent RNA quantification, measured by qRT-PCR, of GR target genes (1 hour of EtOH or dexamethasone treatment) after 48 hours of siRNA transfection against nontargeting sequence (siCTRL) or NIPBL. Fold inductions (dexamethasone/EtOH) are normalized on glyceraldehyde-3-phosphate dehydrogenase (GAPDH) nascent RNA. Error bars represent SE, and *P* value was derived from unpaired Mann-Whitney tests (*n* = 3 independent biological replicates). ns, not significant.

The cohesin complex, NIPBL, and TFs are all jointly involved in the modulation of the long-range chromatin interactions (*2*). To assess whether NIBPL regulates GR-bound long-range interactions, we performed GR-HiChIP in NIPBL knockdown cells (Fig. 2, A to C) (*28*). As expected, given that the GR binds mostly at distal regulatory elements, most of the chromatin interactions bound by GR are enhancer-enhancer interactions and enhancer-promoter interactions (Fig. 2D), while only 2.5% are promoter-promoter interactions. Very few GR-bound interactions engage TAD boundaries marked by the presence of CTCF, suggesting that the GR-bound interactions are independently formed from the structural interactions defined at CTCF TAD boundaries (Fig. 2D). Loss of NIPBL reduced both the number and the strength of GR-bound long-range interactions, identified by FitHiChIP (Fig. 2, B to D, and fig. S3, A to C) (*29*). As a control, we performed GR-HiChIP experiments in ethanol (EtOH)–treated cells, where GR is mostly cytosolic, finding no significantly detectable interactions (Fig. 2, B to D). These data indicate that NIPBL modulates GR binding to chromatin loops. To examine whether NIPBL regulates GR-mediated RNA transcription, we quantified nascent RNA transcripts for well-known GR target genes in NIPBL knockdown cells. The genes altered in NIPBL knockdown cells are cobound by both GR and NIPBL after dexamethasone treatment (fig. S1, H and I). These experiments revealed a profound dysregulation of GR-directed gene expression (for both activated and repressed genes) in the knockdown cells, suggesting that NIPBL is essential for proper GR-mediated transcriptional activity (Fig. 2G and fig. S3, D to F). Perturbation of the cohesin complex could result in several indirect effects including changes in cellular morphology and cellular viability. To rule out these secondary effects, we have repeated the siRNA treatment against NIPBL and SMC1a and checked for those potential indirect effects. We did not observe any drastic changes in either morphology or viability, suggesting that the residual SMC1A or NIPBL remaining (after siRNA treatment) in our breast adenocarcinoma cells is enough to maintain our cells in healthy conditions (fig. S3, G and H).

### Ultradeep Micro-C sequencing shows that NIPBL and GR mediate long-range chromatin interactions

**Fig. 3.**
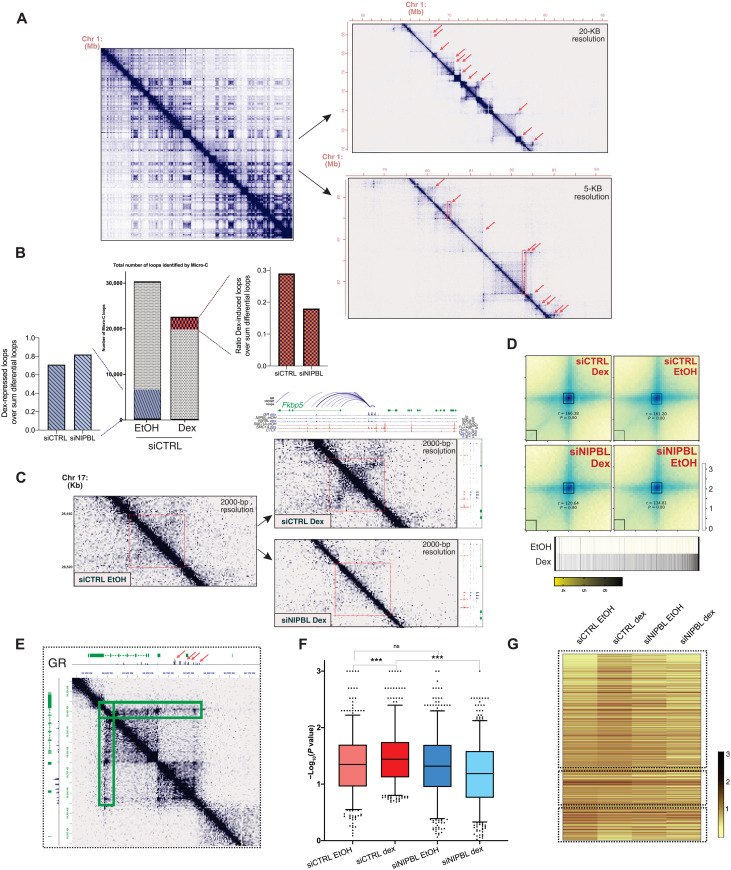
Ultradeep Micro-C sequencing shows that NIPBL regulates GR-mediated long-range interactions. (**A**) Chromosome contact maps for three successive zoom-ins across mouse chromosome 1 using Micro-C data. Red arrows depict anchors by moustache loop calling, and red boxes are architectural stripes detected by Stripenn. (**B**) Middle: Total numbers of Micro-C loops in siCTRL EtOH and siCTRL Dex. A total of 2650 loops were identified as dex-induced while 6350 were found as dex-repressed loops. siNIPBL cells showed a reduction of the dex-induced loops: measured by the ratio of the dex-induced loops over the sum of the differential loops and found by comparing siCTRL EtOH versus siCTRL Dex and siNIPBL EtOH versus siNIPBL Dex. There was no change in the dex-repressed loops as measured by the ratio of the dex-repressed loops. (**C**) Micro-C robustly captures enhancer-promoter contacts driven by the GR. GR, SMC1A, NIPBL, and CTCF ChIP-seq are on top of the contact maps. All normalized datasets represent two biological replicates and plotted at 2-kb resolution. (**D**) Aggregate peak analysis plots of the dex-induced loops. The heatmaps below show the GR ChIP-seq intensity at the dex-induced interactions. (**E**) Contact frequencies, in siCTRL Dex, of the architectural stripe connecting the GR-bound superenhancer to the gene *Nsmce2*. (**F**) GR and NIPBL promote the formation of dexamethasone-dependent architectural stripes. Boxplot depicting the significance [−log_10_(*P* value)] of all the GR-bound architectural stripes (*n* = 561), in siCTRL EtOH, siCTRL dex, siNIPBL EtOH, and siNIPBL dex Micro-C data. (**G**) Heatmap showing the intensity of the architectural stripes (two biological replicates were combined on the level of binned data).

To further understand how GR and NIPBL mediate long-range interactions, we performed Micro-C in two biological replicates (before and after 100 nM dexamethasone) in control and siNIPBL cells. We opted for Micro-C since this method offers great advantages compared to in situ Hi-C, given that the use of micrococcal nuclease provides unparallel resolution (Fig. 3A) (*30*, *31*). After filtering for low-count reads across replicates, we identified 29,674 loops in siCtrl control cells. Dexamethasone treatment resulted in an overall loop count of 22,573. In this fraction, 2650 interactions (12%) represent new loops induced by dex, while 6404 interactions (22%) were eliminated by dex treatment (Fig. 3, B and C, and table S5). Approximately 50% of the new loops created by dex were sensitive to siNIPBL knockdown (Fig. 3B), while very few of the dex-repressed loops were affected by siNIPBL knockdown (Fig. 3B), confirming the importance of NIPBL regulating nuclear architecture (Fig. 3B) (*7*, *14*). Dex-induced interactions are strongly bound by GR, as detected by ChIP-seq (Fig. 3D). The strong reduction of dex-induced interactions in siNIPBL cells indicates that GR and NIPBL jointly promote chromatin looping in a large fraction of the new loops (Fig. 3, B to D). Since the percentage of dex-repressed interactions was not altered by NIPBL knockdown (Fig. 3B), it appears that dex-repressed loops are not regulated by NIPBL. Intriguingly, GR may regulate looping repression through novel cofactors identified in our GR ChIP-SICAP analysis, such as HP1-alpha (Cbx5), HP1-gamma (Cbx3), WIZ, EHMT1 (GLP), lamin B receptor, and nuclear receptor corepressor 1 (table S1).

Among the significant dex-induced loops, our analysis identified chromatin interactions connecting GR-bound enhancers to the GR target genes Fkbp5 and Tsc22D3 (Fig. 3C and table S5) (*24*). NIPBL and SMC1A bind these enhancer-promoter contacts in a dexamethasone-dependent manner (Fig. 3C). These interactions are strongly impaired by the loss of NIPBL (Fig. 3C). The TAD boundaries at these loci are already formed before dexamethasone treatment; however, the activation of the GR induces both the formation of enhancer loops and mostly of the architectural stripes connecting the GR site to the TAD boundaries (Fig. 3E and fig. S2F). Architectural stripes are associated to the loop extrusion process (*13*), where a loop anchor interacts with the entire domain at high frequency, to activate target gene activation (*13*, *32*). To accurately identify these features, we used the Stripenn protocol (*32*) to analyze our Micro-C data (Fig. 3E). Our analysis, at 5000–base pair (bp) resolution, identified 2300 stripes in control EtOH versus 1703 in control dexamethasone cells. NIPBL knockdown lowered the numbers of stripes to 1157 versus 557 (table S6). A total of 561 architectural stripes (at 5-kb resolution) were specifically bound by the GR (table S6). Dexamethasone treatment significantly strengthened the GR-bound stripes compared to the EtOH control, suggesting that GR chromatin binding promotes a higher frequency of interactions and stimulates the loop extrusion process (Fig. 3, F and G). This dexamethasone-induced activation of the architectural stripes is lost after NIPBL knockdown. Together, these data suggest that GR and NIPBL promote DNA extrusion through the formation of both enhancer-promoter contacts and architectural stripes.

### Single-molecule nascent RNA-FISH reveals that GR associates with NIPBL to promote long-range gene regulation

**Fig. 4.**
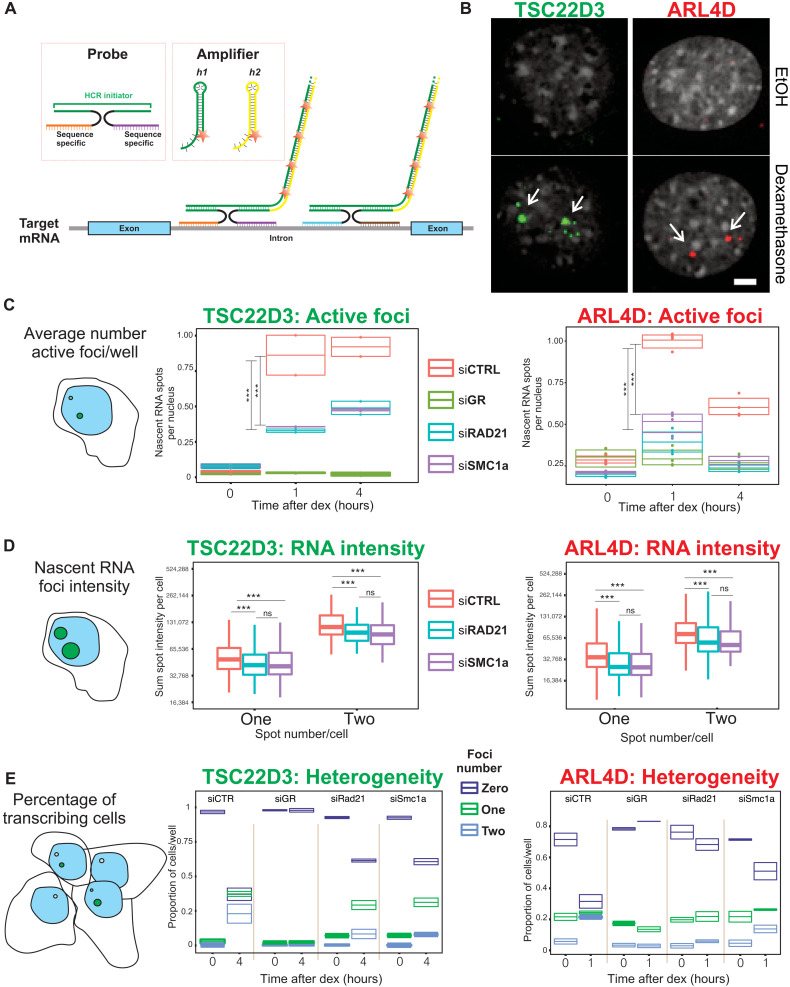
Cohesin depletion impairs GR gene activation. (**A**) Schematic illustration of HCR protocol. (**B**) Representative images of nascent RNA before and after Dex treatment. ARL4D images are taken after 1 hour of dexamethasone; TSC22D3 images are taken after 4 hours of Dex treatment. Scale bar, 5 μm. (**C**) Average number of active nascent RNA foci per well at 0, 1, and 4 hours of dex treatment for ARL4D and TSC22D3, after 72 hours of siRNA against SMC1a or RAD21. Left: TSC22D3; right: ARL4D. TSC22D3 *P* value is 0.012 for siRAD21 and 0.0005 for siSMC1a, both derived from two-way analysis of variance (ANOVA) test. ARL4D, *P* < 0.00001 derived from two-way ANOVA test both for siSMC1a and siRAD21. (**D**) Nascent RNA intensity of each of the foci in control cells and SMC1a and RAD21 knockdown cells. Left: TSC22D3; right: ARL4D. *P* < 0.00001 for TSC22D3 and *P* < 0.001 for ARL4D, derived from two-way ANOVA test for siSMC1a and siRAD21. No statistically significant difference was found between siSMC1a and siRAD21 cells. (**E**) Percentage of actively transcribing cells measured by HCR before and after dex treatment in control and SMC1 and RAD21 knockdown cells. Left: TSC22D3; right: ARL4D. At 0-hour treatment, almost 100% of cells are not actively transcribing. After 1 or 4 hours of dexamethasone treatment, approximately 70% of control cells transcribe nascent RNA of the target genes. In siSMC1a or siRAD21 cells, only 30% of cells actively transcribe nascent RNA for GR-regulated genes. siGR cells, the negative control, show no gene activation after dexamethasone treatment, confirming that nascent RNA foci enrichment is GR dependent.

To accurately investigate gene expression changes upon loss of cohesin at the single-cell level, we used a high-throughput version of hybridization chain reaction (HCR) RNA fluorescence in situ hybridization (FISH) to probe the expression of nascent RNA from well-known GR target genes Tsc22d3 and Arl4d (Fig. 4, A and B). The results confirm that nascent transcription of both Tsc22d3 and Arl4d genes is impaired approximately two- and fourfold in siRAD21 and siSMC1a knockdown cells, respectively (Fig. 4, C and D, and fig. S4, A to D). With HCR, one can investigate the distribution of gene expression at the single-cell and single-allele level. Thus, we measured the percentage of actively transcribing cells for each condition and their allelic contribution (Fig. 4E). Approximately 65% of cells treated with a scrambled siRNA actively transcribe the target genes examined, while in both siSMC1a- and siRAD21-treated cells, less than 40% of these are transcriptionally active (Fig. 4E). Considering the function of the cohesin complex in nuclear architecture, we also investigated whether nuclear positions of the loci are affected. We analyzed the localization of Tsc22d3 and Arl4d loci (measured by HCR) with respect to the nuclear membrane, observing a recruitment of active loci to the periphery of the nucleus under all the tested conditions, while finding no difference between control and knockdown cells (fig. S4E). Collectively, our single-molecule nascent RNA-FISH data show that the loss of NIPBL and of the core cohesin subunits markedly affects the GR-mediated gene expression by reducing the number of bursting sites and the intensity of the transcriptional activity without affecting localization of target genes in the nucleus.

### Acute loss of cohesin impairs inducible TF binding to chromatin

**Fig. 5.**
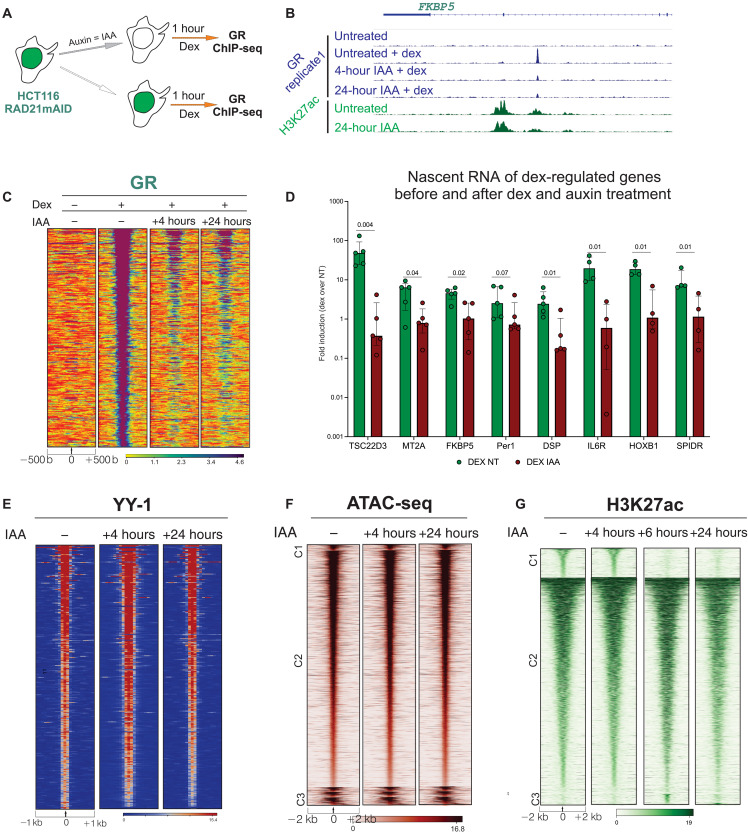
Cohesin acute depletion impairs GR binding to chromatin. (**A**) Schematic representation of ChIP-seq experiments after double treatment of dexamethasone and auxin of the HCT116 RAD21mAID cells. (**B**) Genome browser screenshot of representative GR-bound chromatin location in HCT116 RAD21mAID cells. Two biological replicates are combined for each sample. (**C**) Heatmap representing GR ChIP-seq intensity at GR chromatin–bound locations (*n* = 371) identified in HCT116 RAD21mAID cells. Data combine two independent biological replicates. (**D**) Nascent RNA quantification before and after dexamethasone treatment before and after 6 hours of auxin treatment in RAD21mAID cells. Log_10_ fold inductions (dexamethasone/EtOH) are normalized on GAPDH mRNA. Five biological replicates are shown for each column. Columns depict mean with error bars representing SD between experiments. *P* values derived from unpaired Mann-Whitney-Wilcoxon test. (**E** to **G**) YY1, H3K27ac ChIP-seq, and ATAC-seq in RAD21mAID cells, before and after auxin treatment. No substantial changes are found in YY1 chromatin binding and chromatin accessibility after acute depletion of cohesin subunit RAD21. Data combine two independent biological replicates each. All heatmaps and genomic data are normalized to a total of 10 million reads and further to local tag density. Homer and bedtools algorithms were used to identify unique and common sites between each experimental condition.

To extend our findings, we used the auxin degron system to rapidly deplete the cohesin subunit RAD21 in HCT116 cells (Fig. 5A and fig. S5, A and B) (*6*, *33*). We performed GR ChIP-seq (two independent biological replicates) after 1 hour of dexamethasone induction in untreated and auxin-treated cells and found that rapid depletion (4 and 24 hours) of RAD21 drastically reduces GR binding to chromatin (Fig. 5, B and C). At the RNA level, the acute depletion of RAD21 strongly impaired GR-induced transcription of TSC22D3 and several other target genes (Fig. 5D).

We further asked whether loss of cohesin could also impair chromatin binding of a noninducible TF. To this end, we performed ChIP-seq for YY1, a noninducible TF known to bind and regulate distal regulatory elements. We found that YY1 binding was largely unaffected after 24 hours of auxin treatment, confirming that the cohesin complex depletion has little impact on this stably bound TF (Fig. 5E). To explore whether overall chromatin accessibility and histone acetylation were altered by the acute depletion of RAD21, we performed ATAC-seq (assay for transposase-accessible chromatin using sequencing) (*34*) and H3K27ac ChIP-seq before and after auxin treatment (two independent biological replicates). In agreement with previous studies (*6*), acute depletion of RAD21 (4 to 24 hours) showed minor changes in chromatin accessibility and histone acetylation (Fig. 5, F and G), indicating that the steady-state transcriptional machinery is largely unaltered by the acute loss of RAD21. Nevertheless, prolonged cohesin deprivation (72 hours) leads to the loss of active chromatin modifications and cohesin-associated proteins (fig. S5, E and F).

**Fig. 6.**
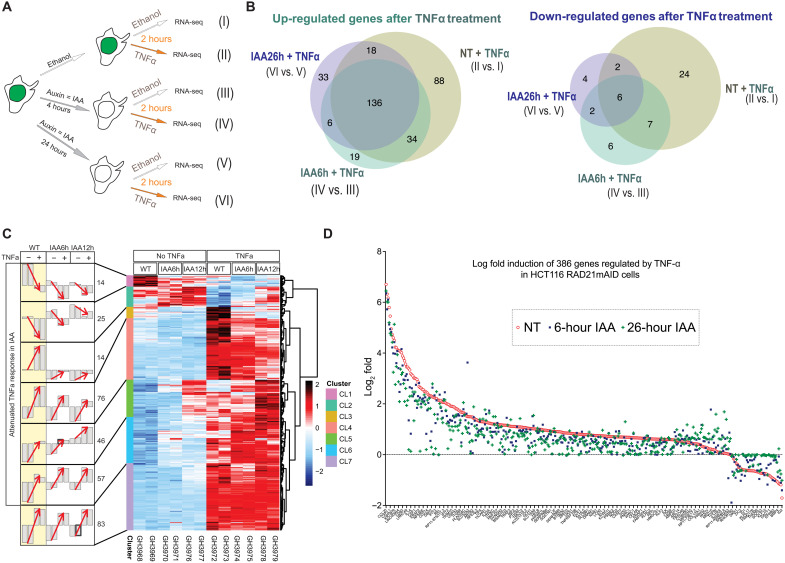
NF-kB response to TNF-α treatment is weakened after cohesin loss, suggesting that inducible TFs promote gene expression through the cohesin complex. (**A**) Schematic representation of RNA-seq experiments after double treatment of dexamethasone and auxin of the HCT116 RAD21mAID cells (*n* = 2 independent biological replicates). (**B**) Venn diagram showing up-regulated genes (left) and down-regulated genes (right) after 2 hours of TNF treatment in untreated cells, 6-hour IAA-treated cells, and 26-hour IAA-treated HCT116 RAD21mAID cells (II versus I, IV versus III, and VI versus V). Differentially expressed genes (DEGs) were obtained by DESeq2 using threshold of adjusted *P* < 0.05 and fold change of 1.5. (**C**) Right: Heatmap summarizing 315 DEGs that are the union of (B). Left: Biological duplicates are shown as two individual bars under each condition. The *y* axis of each bar represents the average of *z*-scored values of variance stabilizing transformed gene expression values in each cluster. Red arrows indicate the response to TNFa treatment under WT, IAA6h, and IAA24h based on average expression profiles. CL1 and CL2 show the attenuated suppression response by TNFa under IAA treatment. CL3, CL4, CL5, and CL6 show the attenuated induction response (with various degrees) by TNFa under IAA treatment. CL7 contains genes less affected by IAA. (**D**) Log_2_ fold changes estimated from DESeq2 of TNF-α–regulated genes (*n* = 386) in (B). Genes are sorted on the basis of the fold change values by TNF-α treatment in IAA-untreated cells to compare the trend of attenuated TNF-α treatment response in IAA-treated cells.

To validate our findings with a nonsteroidal, inducible TF, we characterized the response of nuclear factor kB (NF-kB) to tumor necrosis factor–α (TNF-α) stimulation in the RAD21mAID cells (±auxin) by RNA sequencing (RNA-seq; two independent biological replicates). Elegantly, earlier work using the Hct116 RAD21mAID cells showed that the acute loss of the cohesin complex results in genome-wide loss of chromatin loops (*6*). However, 6 hours of acute RAD21 depletion did not significantly alter the steady-state transcriptomic profiles. Here, we investigated whether the acute loss of RAD21 was altering transcriptional events driven by inducible TFs. As predicted, the TNF-α–induced gene response was altered in the auxin-treated cells, supporting our hypothesis that the inducible TF NF-kB requires cohesin to properly induce gene expression (Fig. 6, A to D). Most of the TNF-α up-regulated genes were differentially regulated in both untreated and auxin-treated cells (Fig. 6B), while more than 50% of the TNF-α down-regulated genes were not found in the RAD21-depleted cells. This suggests an important role for cohesin to regulate gene repression. The log-fold induction of all genes regulated by TNF treatment stimulation was impaired after RAD21 depletion (Fig. 6, C and D), suggesting an essential role for the cohesin complex to fine-tune gene activation and gene repression. Supporting our findings, previous elegant studies performed in mouse macrophages treated with bacterial lipopolysaccharides (LPSs) showed how the cohesin complex regulates gene expression during hematopoietic stem cell differentiation and self-renewal (*23*). On the contrary, the loss of CTCF did not alter the gene expression mediated by LPS treatment in human macrophages (*35*), suggesting that cohesin regulates gene expression, independently of the interaction with the CTCF at TAD boundaries.

### Loss of cohesin markedly diminishes nuclear confinement of TFs

**Fig. 7.**
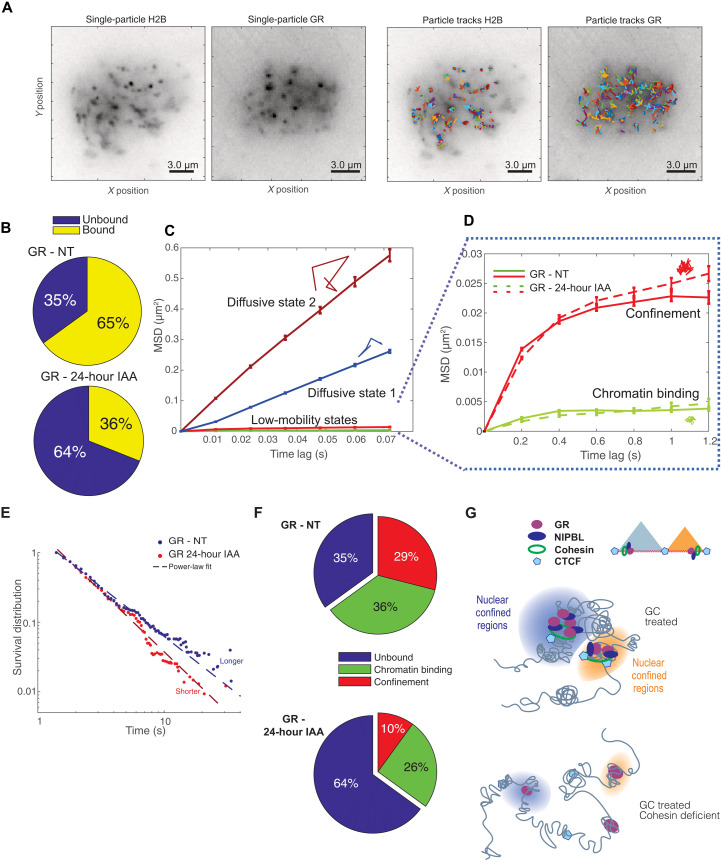
Cohesin mediates TF dynamics and nuclear confinement. (**A**) Time projection of single-particle images from sample SMT experiments after tracking. Left two panels represent the collection of single H2B-Halo and GR-Halo conjugated with JF_549_. Right panels show the collection of trajectories after superresolution localization and tracking. (**B**) Bound versus unbound proportions for GR-Halo before and 24 hours after auxin. To rule out the effects of aberrant mitosis, we imaged only rounded and intact nuclei. (**C**) Mean-squared displacement (MSD) versus lag time for the different diffusivity states detected by pEM for GR-Halo-JF_549_. (**D**) MSD versus lag time for the two bound states of GR-NT (solid line) and GR-IAA after 24 hours of auxin treatment (dashed line). Individual colored tracks in (C) and (D) represent the spatial distribution for each dynamic state. (**E**) Dwell time distribution for the chromatin-bound GR-Halo treated with 100 nM dexamethasone, before (blue dots) and after 24 hours of auxin treatment (red dots). The binding time to chromatin of GR decreases after cohesin depletion (paired KS test, *P* value of 7 × 10^−140^). (**F**) Unbound, chromatin-bound, and confinement fractions of GR-Halo before (top) and after 24 hours of auxin (bottom). The confinement fraction decreases markedly from 29 to 10%, and the chromatin binding fraction decreases from 36 to 26%. Cohesin depletion has a profound effect on the confinement of GR, already noticeable in (D). (**G**) Nuclear confined regions favor long-range chromatin contacts and high concentration of transcriptional regulators. Loss of cohesin results in weaker TF-chromatin interactions at nuclear confined regions.

TFs cooperate with the cohesin complex to modulate long-range gene regulation and nuclear topology. In addition, several studies have investigated the relationship between spatial genome organization and TF biophysics and dynamics (*2*, *12*, *36*). To understand the impact of cohesin on GR dynamics, we used single-molecule tracking (SMT), a powerful superresolution microscopy method to characterize transcriptional dynamics and identify diffusive and chromatin-bound fractions (fig. S7, A to C) (*37*). We performed SMT experiments using the RAD21mAID cell line transfected with Halo-tagged wild-type GR (GRwt-Halo; fig. S7F) visualized by the JF549 fluorophore after activation with dexamethasone (dex). For optimal balance between fast acquisition, highest signal-to-noise ratio, and minimal localization noise, we acquired the image with two different setups: the “fast acquisition,” where the samples were imaged continuously using 12-ms exposure times, and the “slow acquisition,” where images were taken every 200 ms with an exposure of 10 ms. This dual acquisition of the data allows us to precisely quantify the dynamic of GR molecules from diffusing to slower events such as chromatin binding (*36*). The trajectories of localized particles from a representative cell are shown in Fig. 7A.

In agreement with our genomic data, RAD21-depleted cells (using the auxin degron cells) exhibited a strong reduction of GR chromatin interaction compared to the control cells (Fig. 7B). More precisely, the fraction of diffusive (nonbound) GR molecules was increased by 180% after the loss of RAD21 (Fig. 7B). Next, we applied an analysis based on unsupervised machine learning and Bayesian inference criteria (BIC) (*36*, *38*) to classify the molecular trajectories based on their diffusive properties. Using this approach, we observed two categories of diffusive and two categories of bound molecules. We previously reported that the bound category with the most limited movement represents chromatin binding of TFs (green), while the other is associated with nuclear spatial confinement (red) (Fig. 7, C and D, and fig. S7, D and E). The chromatin-bound fraction is determined by the DNA binding properties of the TFs, whereas the confined fraction depends on protein-protein interactions through the TF’s intrinsically disordered region (IDR) (*36*).

Acute loss of RAD21 decreased both the chromatin binding and the nuclear confinement fractions (Fig. 7, E and F). However, the fraction of confined molecules was altered to a much greater extent (Fig. 7F). Loss of cohesin diminishes more than 65% of the nuclear confined molecules of GR (Fig. 7F). Nuclear confined regions have been proposed to arise from highly interacting DNA loops, such as TADs, where a high concentration of TFs promotes dynamic transcriptional events and chromatin intermingling (*3*, *39*). By the formation of these confined regions through their IDRs, TFs could amplify transcriptional output, perhaps by increasing the local concentration of transcriptional regulators at specific chromatin sites (*36*). The real-time microscopy results argue strongly that the cohesin complex is functionally implicated in confinement of TFs (Fig. 7G). These findings imply a novel synergism between TF-mediated long-range interactions, nuclear confined regions, and possibly loop extrusion.

### GC treatment is altered by mutations in the cohesin complex

**Fig. 8.**
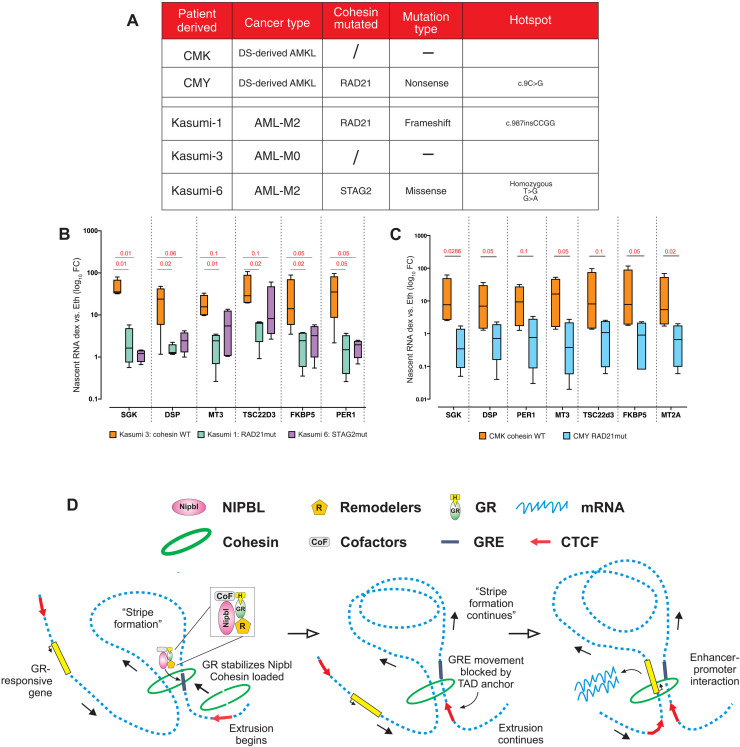
GC treatment depends on the cohesin complex. (**A**) Cohesin mutations in the myeloid leukemia–derived cells used to evaluate GC treatment. (**B**) Nascent RNA quantification measured by qRT-PCR of GR target genes after 2 hours of dexamethasone treatment in AML patient–derived cells: Kasumi-1 (RAD21mut), Kasumi-3 (cohesin WT), and Kasumi-6 (STAG2mut). Log_10_ fold inductions (dex/EtOH) are normalized on β-actin nascent RNA. (**C**) Nascent RNA quantification measured by qRT-PCR of GR target genes after 2 hours of dexamethasone treatment in the DS-AMKL: CMK (cohesin WT) and CMY (RAD21mut). For both (B) and (C), GC treatment is strongly altered in patient-derived cells harboring cohesin mutations. Log_10_ fold inductions (dex/etOH) are normalized on β-actin nascent RNA. Boxplots represent minimum to maximum values, and error bars represent SE (*n* = 4 independent biological replicates). *P* values derived from unpaired *t* test. (**D**) Model of GR-NIPBL synergy to promote cohesin chromatin binding, activation of the loop extrusion, and long-range gene regulation.

Cohesin subunits are often mutated in several types of cancer. In some forms of acute myeloid leukemia (AML), the cumulative level of mutations in *RAD21*, *SMC1a*, *SMC3*, and *STAG2* genes reaches 13% (*40*). This percentage is even higher in Down syndrome–associated AML (DS-AML), where more than 50% of patients harbor cohesin mutations (*40*). To evaluate whether cancer mutations in the cohesin genes can alter the response to GCs, we examined the dexamethasone response with AML patient–derived samples. Kasumi cells and CMK-CMY cells are derived from DS-AML–affected patients (Fig. 8A) (*41*). In both sample sets, the cohesin mutated cells had a much lower response to GC treatment, supporting the model that cohesin modulates GR activity and the GC therapeutic response (Fig. 8, B and C). GCs, such as betamethasone and dexamethasone, are beneficial in leukemia treatment when combined with chemotherapy agents (*42*, *43*), likely due to their anti-inflammatory properties. However, our data suggest that the efficacy of the GC treatment may depend on the integrity of the cohesin complex. When the structure of these proteins is altered because of mutations, GC treatment may be ineffective or even deleterious.

## DISCUSSION

Long-range interactions govern genome organization and function, profoundly altering cellular phenotypes (*1*). TFs modulate chromatin interactions connecting enhancer and promoter to properly modulate cell fate (*2*). Therefore, a clear correlation has been shown between the number and/or strength of chromatin loops and the modulation of transcriptional events (*6*, *9*, *13*). Several studies have investigated this from different perspectives. Phase separation, transcriptional hubs, and TAD heterogeneity are all causes/consequences of the tight relationship between looping and transcriptional machineries (*44*–*47*). The cohesin ring-shaped structure is central to nuclear architecture and is stably bound to chromatin, but its assembly is promoted dynamically by the cohesin loader NIPBL at chromatin accessible sites (*10*, *11*, *13*, *14*, *48*). As NIPBL does not exhibit sequence-specific DNA binding, the mechanism of its recruitment to chromatin remains elusive. TFs, such as steroid receptors, bind inaccessible chromatin, remodel nucleosomes, and create chromatin accessible sites (*26*, *49*). Other studies have shown that steroid hormone receptor activation increases the frequencies of already preestablished loops, and the cohesin complex is important to modulate gene expression driven by steroid receptors (*8*, *21*, *22*, *50*). Here, we demonstrate that site-specific chromatin binding of the NIPBL-cohesin complex occurs at the chromatin accessible sites driven by the activation of TFs such as nuclear receptors (Fig. 1, E and F), promoting chromatin interactions, loop extrusion, and long-range gene regulation.

Subsequently, the cohesin complex extrudes DNA in an ATP-dependent manner toward the nearest TAD boundary (Figs. 1H, 3, F and G, and 8D) (*13*). In this context, our results demonstrate that GR (and most likely other TFs) associates with the NIPBL-cohesin complex, stabilizing cohesin binding at GR-responsive sites, promoting loop extrusion, and strengthening preestablished chromatin loops (Fig. 8D) (*21*, *22*, *24*, *50*). Our work investigated mostly the relationship between the GR and the cohesin loader NIPBL; however, given the interplay between transcriptional regulators and the cohesin complex to regulate genome organization (*2*, *8*, *16*, *17*, *21*), other TFs may follow the same pattern. Nevertheless, some TFs may modulate long-range gene regulation through other mechanisms. Other studies, however, have shown that multiple chromatin regulators, including mediator subunits, chromatin remodelers, and CBP/P300, are associated with the cohesin complex at cohesin-enriched locations, suggesting a multifactorial model where many factors cooperate to regulate long-range gene regulation. However, as for NIPBL, these factors do not recognize specific sites. We demonstrate here that the process is initiated, at enhancers, by TFs.

The association between TFs and the cohesin complex occurs in a positive feedback mechanism, increasing the concentration of TFs in the environment of specific sites, thus amplifying transcriptional output (Figs. 4 to 6). This concept is particularly evident through the SMT experiments, which demonstrate a significant loss of the confinement population of GR upon depletion of the cohesin complex (Fig. 7).

An important discussion in the community concerns whether an active regulatory enhancer must build a specific contact with the promoter or simply migrate into the neighborhood of the promoter (*6*, *51*, *52*). Although the data presented here neither support nor contradict either of these hypotheses, our results point to a mechanism wherein TFs initiate the binding of NIPBL-cohesin to chromatin, mediating the loop extrusion mechanisms that drive the enhancer near the promoter (Fig. 8D). Even if most of the interactions bound by the GR are already present before hormone activation (*22*), our analysis showed dex-induced strengthening of both chromatin loops and architectural stripes (Fig. 3). Our data demonstrate that GR and NIPBL promote the formation architectural stripes, reinforcing our hypothesis that TFs associate with the cohesin complex initiating DNA extrusion to promote long-range gene regulation.

From the clinical perspective, NIPBL and cohesin subunits are frequently mutated in fast-growing cancers such as AML (*40*). However, patients do not harbor mutations in more than one cohesin subunit, suggesting that co-occurring mutations are not advantageous for cancer cells (*40*). Instead, NIPBL is very rarely mutated in cancer. On the other hand, NIPBL is a hallmark of the Cornelia de Lange syndrome, a disease exhibiting growth anomalies, facial dysmorphism, and cognitive retardation (*53*, *54*). Therefore, there is a disconnect between the phenotypes observed by NIPBL mutations (slow growth) and the phenotypic changes observed with the cohesin core subunits (fast growth).

Last, we show that GC stimulation relies on the status of the cohesin complex to properly regulate gene targets in AML cell lines known for their high incidence of cohesin gene mutations (Fig. 8, A to C). Although GCs are extensively studied and characterized, the treatment of respiratory diseases and their potential usefulness in cancer may be marginalized by mutations in cohesin function. NF-kB gene regulation was also hampered by Rad21-depleted cells (*23*), suggesting that cohesin associates to a much greater panel of TFs (Fig. 6) (*16*). Therefore, consideration of the mutational landscape of the patient, especially the presence of cohesin mutations, could help in the evaluation of possible benefits of GC and/or cytokine treatment.

## MATERIALS AND METHODS

### Cell culture, auxin treatment, and siRNA transfection

Cells of 3134 mammary breast carcinoma were grown in Dulbecco’s modified Eagle’s medium (DMEM) and 10% fetal bovine serum (FBS) supplemented with sodium pyruvate, l-glutamine, and nonessential amino acids. HCT116 RAD21mAID cells were grown in McCoy medium supplemented with 10% charcoal-stripped FBS (CSS) and l-glutamine. Complete RAD21 depletion was achieved by the addition of 500 μM indole-3-acetic acid (IAA; from Millipore/Sigma-Aldrich). AML cells were grown in RPMI medium supplemented with 20% FBS, pyruvate, l-glutamine, and essential amino acids. Before each dexamethasone (100 nM) treatment experiment, all cells were grown for 48 hours in CSS. To achieve an effective knockdown, 5 million 3134 cells were transfected during log-phase growth, by electroporation (140 V, 10 ms, three pulses) with 10 μg of each siRNA against NIPBL, SMC1, or RAD21. Smartpool siRNAs were purchased from Dharmacon. Cells were treated with vehicle or dexamethasone after 48 and 72 hours after electroporation. After vehicle or dexamethasone treatment, nascent RNA was extracted using the Macherey-Nagel Kit following the manufacturer’s instructions. Reverse transcription was carried out using 1 μg of RNA using the Bio-Rad complementary DNA synthesis kit. qPCR was performed using Bio-Rad SYBR-Green Master Mix.

### Chromatin immunoprecipitation–selective isolation of chromatin-associated proteins

The procedure basically follows the initial publication with some adjustments (*25*). A total of 25 million mammary breast carcinoma cells were cultured for each condition and treated with corticosterone (600 μM) or EtOH for 1 hour. Cells were then fixed for 13 min with 1% paraformaldehyde, washed three times with phosphate-buffered saline (PBS), and collected. After 1 hour of incubation with lysis buffer [0.5% SDS, 10 mM EDTA, and 50 mM tris-HCl (pH 8.1)], cells were sonicated to reach a 500-bp shredded chromatin (Bioruptor, Diagenode). Chromatin was then diluted and incubated overnight at 4°C with GR antibody preconjugated beads (sc-393232, Santa Cruz Biotechnology; MA1-510, Thermo Fisher Scientific). On-beads chromatin was then tagged with ddUTP-biotin (NU-1619-biox, Jena Bioscience) using a DNA terminal transferase for 1 hour at 37°C (M0315, NEB). Beads were then successively washed with low-salt, high-salt, and LiCl buffer, and the chromatin was eluted for 15 min at 37°C in the SICAP elution buffer [7.5% SDS and 200 mM dithiothreitol (DTT)]. Chromatin was then resuspended in SICAP buffer [0.5% NP-40, 1% Triton X-100, 5 mM EDTA, 50 mM tris-HCl (pH 7.4), and 150 mM NaCl] and incubated with streptavidin beads (NEB) for 1 hour at room temperature. The beads were then extensively washed with the following solutions: three times with the SDS buffer [1% SDS, 1 mM EDTA, 10 mM tris-HCl (pH 7.4), and 200 mM NaCl], then once with the BW2x buffer [0.1% Triton X-100, 1 mM EDTA, 10 mM tris-HCl (pH 7.4), and 2 M NaCl], then twice with 20% isopropanol, and, lastly, four times with 40% acetonitrile. Dry beads were then frozen at −80°C and sent for MS analysis.

### Proteomics

#### GR ChIP-SICAP spectral library generation and data-dependent acquisition

All proteomic experimental procedures were already described in detail in our previous work (*55*). In brief, a fraction (20%) of trypsin-digested peptides from each ChIP-SICAP–obtained samples were combined and used to generate the spectral library for the following liquid chromatography–tandem MS (LC-MS/MS) data-independent acquisition (DIA). The combined peptide mixture was fractionated using high-pH reversed-phase chromatography on an Ultimate3000 high-performance liquid chromatography system (Thermo Fisher Scientific) using a 10-cm-long ACQUITY CSH C18 1.7-μm column (Waters). The fractionated peptides, together with the input flow-through fraction, were vacuum-dried in a speed-vac, resolubilized in 9 μl of 0.5% AA in water, and used for the nanoLC-MS/MS analysis on a Q Exactive HF-X mass spectrometer coupled with an EASY-nLC 1000 ultrahigh-pressure system (Thermo Fisher Scientific). MS data were acquired using a data-dependent acquisition (DDA) method switching between full scan events and the top 12 MS/MS scans. An automatic gain control target value was set to 3 × 10^6^, and resolution was set to 60,000 for full MS scan events with a scan range of 300 to 1700 mass/charge ratio (*m/z*) and a maximum ion injection time (IT) of 15 ms. Precursors were fragmented by higher-energy collisional dissociation with a normalized collisional energy of 28%. MS/MS scans were acquired with a resolution of 60,000, maximum IT of 110 ms, and 1.2 *m/z* isolation window. The obtained Thermo .raw files were analyzed using MaxQuant software (version 1.5.2.8, Max Planck Institute of Biochemistry, Martinsried, Germany) and a *Mus musculus* FASTA file downloaded from UniProt (www.uniprot.org/) in January 2019, supplemented with commonly observed contaminants. The MaxQuant search settings for maximum missed cleavages were set to 2, peptide mass tolerance to 4.5 parts per million (ppm), and fragment ion tolerance to 20 ppm, and trypsin was chosen as enzyme. Variable modifications were specified to include oxidation on methionine and acetylation on protein N-terminus. As fixed modification, carbamidomethylation of cysteine was specified. MaxQuant data were filtered for reverse identifications, with false discovery rate (FDR) set as 1%.

#### DIA and data processing

After generating a GR ChIP-SICAP spectral library using a combination of DDA .raw files from method test runs (*n* = 6) and high-pH fractionations (*n* = 14), the remaining 80% of trypsin-digested peptides from each ChIP-SICAP sample were analyzed using a DIA method exactly as described in our previous works (*55*). DIA raw files were analyzed using Skyline v4.2.0.18305 (MacCoss Lab software, University of Washington) following the authors’ guidelines and settings. Library ion match tolerance was set to 5 mDa, and MS/MS filtering was set to centroid with a 10-ppm mass accuracy. Retention time (RT) filtering was set to use only the scan within 15 min of the predicted RT and to extract the area under the curve relative to the five most intense product ions for each peptide. mProphet peak scoring algorithm was trained against the decoy peptide library and used to identify correctly integrated target peptide with a *Q* value of <0.01 (i.e., 1% FDR). Data matrix was exported in .csv format, and subsequent analysis of data was performed in Excel. In brief, after normalization using the median of MS/MS intensities within the runs, differential expression analysis was performed using only proteins identified in at least 50% of the samples and with a fold change of ≥2 among the different GR treatment conditions. Missing values were filled by randomly picking a number in the 1% percentile of the distribution of each condition of each replicate. Significant GR interactors at chromatin level are listed in table S1.

#### High-throughput HCR

Appropriate siRNA oligos (0.25 pmol) were spotted at the bottom of each well of a 384-well imaging plate (6057300, Greiner) using an Echo525 (Beckman Coulter) acoustic liquid handler. The siRNA oligos were air-dried, and the plates were then sealed and stored at −20°C. The day of the transfection, plates were thawed and spinned down at 500*g* for 1 min. For each well, dried oligo siRNAs were resuspended in 20 μl of Opti-MEM (Thermo Fisher Scientific) containing 50 nl of RNAiMax (Thermo Fisher Scientific) for 30 min at room temperature. A total of 500 mammary breast carcinoma cells were added to the complexed siRNA/transfection mix in 20 μl of culture media containing 2× serum, for a total volume of 40 μl, and cultured for 72 hours at 37°C. Cells were then processed for HCR according to a slightly modified version of the original protocol (*56*). Briefly, after the specified treatment (+10 μl), imaging plates were fixed by adding 50 μl of 8% paraformaldehyde in PBS directly to the cells, incubated at room temperature for 20 min, and then washed for three times with PBS using a Bluewasher plate washer (Blue Cat Bio). Last, cells were permeabilized with 70% EtOH at −20°C for a minimum of 8 hours. Cells were rehydrated with 5× SSC Tween 0.1% buffer (5× SSCT) and preincubated with the hybridization buffer (Molecular Instruments) for 30 min at 37°C. Custom-design probes targeting intronic regions of the specified target genes Tsc22d3, Arl4d, Ccl2, and Cxcl5 (Molecular Instruments) were then added at a final concentration of 2 nM in a 10-μl volume using a Mosquito liquid handler (SPT-Labtech) and incubated overnight at 37°C. Probes were later washed for 15 min at 37°C with the Probes Wash buffer (Molecular Instruments) with increasing proportions of 5× SSCT (25, 50, and 75%) then washed twice with 100% 5× SSCT (first at 37°C and second at room temperature). Later, probes were preincubated with the Amplifier buffer (Molecular Instruments) and then incubated with 75 nM proper hairpin amplifiers for 45 min at room temperature. Wells were next washed three times for 20 min each with 5× SSCT at room temperature. Following that, cells were stained with 4′,6-diamidino-2-phenylindole (DAPI) for nuclear localization, and images were acquired on a CV7000 high-throughput microscope (Yokogawa). Images analysis was lastly performed on the Columbus platform then with a custom R script.

#### Proximity ligation assay

Mammalian breast cancer cells were plated in 386-well plates (MGBB096-1-2-LG-L, Matriplate, Brooks), and proximity ligation assay was performed following the manufacturer’s instructions (DUO92101, Sigma-Aldrich). Briefly, after treatment, cells were fixed with 4% paraformaldehyde for 20 min and then washed extensively with PBS. Next, permeabilization was achieved with a PBS solution with 0.5% Triton X-100 for 20 min, and cells were blocked with PBS with 3% bovine serum albumin and 0.05% Triton X-100 solution for an hour at room temperature. A second blocking was performed with the manufacturer’s solution for another hour at 37°C. After antibody incubation (see the “Antibodies” section; room temperature for 45 min), probes were added and incubated for an hour at 37°C, washed with buffer A, ligated (30 min at 37°C), and washed again with buffer A. Last, signal amplification was accomplished for 100 min at 37°C and washed with buffer B before getting stained with DAPI. The images were then acquired on a CV7000 microscope (Yokogawa) and analyzed on the Columbus platform.

#### Subcellular fractionation

About 2 million 3134 cells were detached by Accutase digestion, then centrifuged (5 min, 300*g*, 4°C), and lastly washed three times with ice-cold PBS. Cells were then incubated on ice for 10 min with buffer A [15 mM tris-HCl (pH 8.0), 15 mM NaCl, 60 mM KCl, 1 mM EDTA, 0.5 mM EGTA, and 0.02% NP-40] to extract the cytoplasmic fraction by centrifugation at 1300*g* for 5 min at 4°C (supernatant). The purified nuclei (pellet) were then washed twice with buffer A and rotated for 1 hour at 4°C with buffer B (3 mM EDTA, 0.2 mM EGTA, and 1 mM DTT). Following that, the samples were centrifugated (5 min, 1400*g*, 4°C), and the chromatin fraction was collected (insoluble). The pellet of chromatin was then washed twice with buffer B, and the proteins were extracted with 50 mM tris (pH 8), 1 mM EDTA, 0.05% SDS, and 250 U of benzonase in the presence of 2 mM MgCl_2_ for 1 hour. After centrifugation (10 min, 15,000*g*, 4°C), the supernatant (chromatin fraction) was lastly collected. Equal amounts of protein from the different fractions were then processed for the Western blot procedure.

#### Immunoprecipitation

About 20 million cells were treated with EtOH or dexamethasone (100 nM) and fixed for 12 min with 1% paraformaldehyde. After three washes with PBS, cells were harvested and incubated with the lysis buffer (see ChIP-SICAP procedure) on ice for an hour. After sonication, to obtain an average DNA length of 500 bp, the lysate was diluted and incubated overnight at 4°C with GR antibody preincubated beads (sc-393232, Santa Cruz Biotechnology; MA1-510, Thermo Fisher Scientific). The next day, the beads were washed successively with low-salt, high-salt, LiCl, and lastly TE buffer. Then, the beads were incubated in 1× Laemmli buffer for 10 min at 95°C for elution/denaturation and loaded on a precast 3 to 8% tris-acetate gel (Thermo Fisher Scientific). Proteins were next transferred on a polyvinylidene difluoride membrane for Western blotting.

#### ChIP-seq and ATAC-seq

Duplicate biological replicates were carried out for each ChIP-seq and ATAC-seq experiment. For GR ChIP-seq, 10 million to 20 million log-phase growth cells were treated with vehicle (EtOH) or treated with 100 nM dex (Sigma-Aldrich) for 1 hour. For ChIP, after cross-linking with paraformaldehyde and cell collection, the chromatin was sonicated (Bioruptor, Diagenode) to an average DNA length of 200 to 700 bp. For immunoprecipitation, 1000 μg of chromatin was incubated with appropriate antibody coupled onto Dynabeads magnetic beads (Thermo Fisher Scientific) with rotation overnight at 4°C. Then, the chromatin-bead conjugates were washed with low-salt, high-salt, and LiCL buffer and eluted in 1% SDS and 100 μM NACO_3_, proteinase K–treated, and the cross-linking was reversed at 65°C for 7 hours. DNA was extracted from the samples with phenol-chloroform extraction and EtOH precipitation. ChIP-seq libraries were generated using a TruSeq ChIP sample prep kit (Illumina, IP-202-1012) according to the manufacturer’s instructions. For NIPBL ChIP, we implemented a disuccinimidyl glutarate (DSG)/FA cross-link that has been shown to allow efficient detection of weaker NIPBL-binding sites (*57*). Briefly, cells were suspended in PBS and treated for 45 min with 2 mM DSG. After three washes with PBS, cells were cross-linked with formaldehyde as described above. For ATAC-seq, the cells were detached from the flasks using 5 ml of Accutase (Thermo Fisher Scientific) by incubating for 5 min at room temperature. ATAC was performed according to Omni-ATAC protocol with double amount of transposase compared to the original protocol. Size selection was performed using SPRIselect (Beckman Coulter) to remove <150-bp and >1000-bp fragments according to the manufacturer’s instructions. Size selection was verified using the Agilent TapeStation System (Agilent Technologies).

#### ChIP and ATAC-seq analysis

Biological duplicates were sequenced using Illumina NextSeq 500 single reads, whereas ATAC-seq was sequenced pair-ended. The reads were trimmed in silico to remove adapter sequences, low-quality reads, and 50-bp length using Trimmomatic 0.30 software and aligned to mm10 reference genome using Bowtie2 alignment tool. Mitochondrial reads were filtered for the subsequent analyses. All peak calling of ATAC-seq data was performed using MACS2 v.2.1.1 with callpeak-format BAMPE parameters. The heatmaps were generated using an in-house R script.

Data were aligned to the mouse reference mm10 genome by STAR (*58*). ChIP-seq was analyzed mostly using homer (*59*) and bedtools. Cohesin quantification at previously identified GR sites was performed by homer using annotatePeaks.pl -ghist. siSMC1a and siNIPBL GR peaks were identified using homer findpeaks algorithm using style for TF and style histone for H3k27ac. Input DNA was used as control sequence. Genomic results such as cohesin enrichment after dexamethasone treatment are listed in table S2.

#### Micro-C

Duplicate biological replicates were carried out for each Micro-C experiment, following the published protocol (*30*, *31*). Briefly, after evaluating the best conditions for MNase treatment (nuclei preparation using Igepal at 0.03% and 200 U of MNase enzyme for 10 min at 37°C) in our mouse breast adenocarcinoma cells, we performed all Micro-C protocol as described in detail in (*30*, *31*) using 2.5 million 3134 mouse breast adenocarcinoma cells for each biological replicate. Briefly, after stopping the MNase reaction for 10 min at 65°C, the DNA ends were dephosphorylated using rSAP for 45 min at 37°C. Later, the 5′ overhangs were generated by 3′ resection by adding Klenow fragment polymerase and PNK in a nucleotide-free solution for 15 min at 37°C. The DNA overhangs were filled with biotinylated nucleotides for 45 min at room temperature using biotin-ATP, biotin-CTP, 2′-deoxyguanosine 5′-triphosphate, and 3′-deoxythymidine 5′-triphosphate. After stopping the reaction and isolating the nuclei by centrifugation, we incubated the isolated nuclei for 3 hours at room temperature with 12,500 U of NEB T4 ligase. After proximal ligation, nonligated biotinylated ends were removed by incubating the isolated nuclei with 200 U of exonuclease III for 5 min at 37°C. Samples were decross-linked for 7 hours at 65°C with shaking, and DNA was isolated by phenol-chloroform extraction. The 300- to 400-bp-sized (dinucleosome band) Micro-C library was purified by 2% agarose gel (run at 70 V) and purified using the Zimo DNA Clean and Concentrator Kit. Purified dinucleosome samples were quantified using the Qubit DNA HS Kit and balanced among samples. Then, purified DNA was incubated with 5 μl of MyOne C1 streptavidin beads for 20 min at room temperature. After washes, Micro-C sequencing libraries were generated using the Kapa HyperPrep Kit from Roche. To reach high resolution, we sequenced around 3 billion to 4 billion reads for each biological sample using the Illumina NovaSeq sequencer.

#### Micro-C analysis

Micro-C datasets were analyzed following the published bioinformatic pipelines (*30*, *31*). Briefly, raw data were analyzed through the distiller pipeline (*60*) (https://github.com/mirnylab/distiller-nf), mapping to the mouse reference assembly mm10 using bwa mem with the -SP flags. Pairtools package was then used to parse alignments and classify pairs to generate pair files compliant with the 4DN and deduplicated pair files with an option -max-mismatch=1 on either side. Pairs classified as uniquely mapped with high mapping quality scores (MAPQ > 30 for both) were used to obtain contact matrices in the cooler format at 500 bp and in balanced multiresolution cooler format files (500 bp, 1 kb, 2 kb, 5 kb, 10 kb, 20 kb, 50 kb, 100 kb, 250 kb, 500 kb, 1 mb, and 10 mb) using cooler with default options [low-coverage bins were excluded using the MADmax (maximum allowed median absolute deviation) filter on genomic coverage, described in (*30*) using a threshold of 5.0 MADs].

HiC balanced files were generated by cooltools (https://github.com/mirnylab/cooltools) and visualized by Juicebox (*61*). Loop calling and the detection of differential loops between dex-treated and EtOH cells (siCTRL and siNIPBL) were performed by diff_mustache.py function in Mustache (*60*) with default parameter options (loop call FDR = 0.2, differential loop detection FDR = 0.1) except sparsityThreshold (-st) as 0.7.

#### Micro-C Stripenn analysis

To identify the architectural stripes found in our Micro-C dataset, we used the mcool datasets to the compute function of the Stripenn algorithm (*32*) with default parameters (KR normalization, -p 0.2 and -m 0.95,0.96,0.97,0.98,0.99) set at 5000-bp resolution. To identify the GR-bound stripes (*n* = 561), we used bedtools to intersect the Stripenn-identified architectural stripes with our GR ChIP-seq peaks. Then, we used the Stripenn score function to calculate the significance (*P* value) of the GR-bound architectural stripes in all our sample conditions, at 5000-bp resolution, using default parameters (*32*).

#### CTCF boundary prediction

CTCF boundaries were predicted using the script published by Oti *et al.* (*27*) run on CTCF ChIP-seq peaks published previously in mouse breast adenocarcinoma 3134 cells (*24*). Briefly, we overlapped the genome-wide CTCF motifs with the CTCF peaks to obtain the subset of peak motifs. We used homer to quantify CTCF peaks, and we considered for the follow-up analysis only the CTCF peaks above 40 normalized sequenced tag. Peak-contained motifs were considered as possible anchors for loop prediction. Two possible anchors constituted a TAD loop when the two CTCF motifs were convergent. The probability and scores of the loop anchors were computed from both peak and motif scores by multiplying the ChIP-seq peak scores. We intersected the predicted TADs (with high probability) with GR ChIP-seq dataset to obtain a list of predicted TADs containing a GR-bound site and a list of TADs with no GR-bound sites.

#### HiChIP

GR-HiChIP was performed following the previously published HiChIP protocol (*28*). Briefly, cells, after dexamethasone treatment, were detached using Accutase (Thermo Fisher Scientific) before cross-linking with formaldehyde for 10 min and quenched with 125 mM glycine. Here, 3134 cells were lysed in preparation for in situ contact generation. Isolated nuclei were permeabilized, and restriction digestion was carried out for 2 hours at 37°C with Mbo I (New England Biolabs). Restriction sites were filled with dNTPs using biotin-14-dATP (Jena Bioscience) for 1 hour at 37°C. The filled ends were then ligated together using a T4 ligase at room temperature for 4 hours before nuclei were lysed and sonicated (using Covaris), and then GR immunoprecipitation was carried out overnight using antibodies. The morning after, 30 μl of beads was added to collect the chromatin-antibody complex; then, the ChIP DNA was collected and washed, and cross-links were reversed overnight using proteinase K. ChIP DNA was eluted, and samples were purified using the DNA Clean up Kit. The DNA was quantified using a Qubit before biotin ligation junction capture using streptavidin C-1 beads. Samples were washed and taken forward for Tn5 tagmentation. Tagmentation and PCR amplification were performed as described by Mumbach *et al.* (*28*). Libraries were size-selected to 200 to 700 bp and sequenced on the HiSeq using 2 × 150 bp. HiChIP fastq files were aligned using HiCPro (*62*), and thereafter, FitHiChIP (*29*) was used to identify significant interactions and interaction strength for each experimental condition. CoverageBias was used to normalize the data and call significant interaction. The genome browser WashU was used to visualize the HiChIP loops. GR-HiChIP significant interaction at *q* value of 0.01 for samples EtOH, siCTRL, and siNIPBL is listed in table S3.

#### Viability assay

A total of 2000 cells treated with the indicated siRNA were plated into a 384-well plate (Greiner). Twenty-four hours later, we applied the Live/Dead Sytox staining procedure recommended by the manufacturer (S1138, Thermo Fisher Scientific). Briefly, cells were stained for 15 min in regular growing medium then washed three times with PBS. The images were acquired on a CV7000 microscope (Yokagawa) and analyzed using the Columbus platform.

#### Single-molecule tracking

Data acquisition of SMT experiments was described previously (*36*, *63*, *64*). Briefly, HCT116 RAD21mAID cells were plated into two-well Lab-Tek chamber slides (Thermo Fisher Scientific, Waltham, MA, USA) in Phenol Red–free DMEM medium containing 10% CSS (Hyclone, Logan, UT) and transfected with a Halo-GR plasmid. Later, cells were treated with 500 μM IAA (from Millipore/Sigma-Aldrich) for 24 hours and, the next day, incubated with 0.25 nM cell-permeable Janelia Fluor 549 HALOTag ligand (JF549) for 20 min. Cells were lastly extensively washed with media and treated with 100 nM dexamethasone or EtOH for 20 min before acquisition.

A custom-built microscope (Optical Microscopy Core facility, LRBGE, NCI) controlled by Micro-Manager software (Open Imaging Inc., San Francisco, CA) was used. It was equipped with a 150× 1.45 numerical aperture objective (Olympus Scientific Solutions, Waltham, MA), a 561-nm laser (iFLEX-Mustang, Excelitas Technologies Corp., Waltham, MA), an acousto-optic tunable filter (AOTFnC-400.650, AA Optoelectronic, Orsay, France), and HILO (highly inclined and laminated optical sheet) illumination microscope. Eight hundred frames of fluorescent images were collected on an EM-CCD camera (Evolve 512, Photometrics) at a rate of 5 Hz with 10-ms exposure time to resolve the confinement and slow bound state, while a rate of 83 Hz with 10-ms exposure time was used to study the mean square displacement.

The particle tracking was performed with the “TrackRecord” software developed in MATLAB (MathWorks Inc.). The procedure is summarized in a previous publication (*63*). The molecules were allowed to move a maximum of four pixels from one frame to the next for the 5-Hz acquisition and six pixels for 83-Hz acquisition; for both conditions, only tracks that were at least two frames long were kept.

#### SMT analysis

An improved method that accounts for photobleaching effects was applied to the dwell time distribution analysis (*64*). Briefly, the dwell time distribution of histone H2B was measured at the focal plane under identical SMT acquisition conditions and then fitted to a triple-exponential model to calculate photobleaching parameters. The dwell time distribution is obtained by calculating the weighted ensemble average distribution of bound times for each diffusive state in different cells in the experiment, and then it is corrected by dividing the exponential component estimated in the H2B dwell time distribution analysis. After photobleaching correction, the dwell time distribution is fitted to a power-law distribution.

Using a machine learning–based classification described previously (*36*), we measured the nuclear mobility of chromatin factors. Specifically, we used short exposure times (10 and 12 ms) to minimize motion blur. Then, images were acquired, and trajectories were generated. Perturbation expectation maximation (pEM) together with BIC was used to classify the trajectories of the protein into the least number of diffusive modes. The posterior probability weighted mean-squared displacement (MSD) for each diffusive state was computed. States with a population of less than 5% of tracks with a higher posterior probability than 0.6 were discarded (*36*).

#### RNA isolation and RNA-seq data analysis

RNA was extracted using the Macherey-Nagel Kit, following the manufacturer’s instructions. RIN scores of all samples were above 7.6. RNA-seq libraries were constructed using the Illumina TruSeq Stranded Total RNA with Ribo-Zero Human/Mouse/Rat library kit according to the manufacturer’s instructions. We sequenced two biological replicates for each condition using Illumina NovaSeq6000 S4 with 150-bp paired-end reads. RTA was used for base calling, and Bcl2fastq was used for demultiplexing. Trimmomatic 0.39 was used to trim for adapters. RNA-seq alignment to human hg19 genome was performed by STAR (*58*) using default parameters with the following modifications: “--genomeDir hg19-150 --outSAMunmapped Within --outFilterType BySJout --outFilterMultimapNmax 20 --outFilterMismatchNmax 999 --outFilterMismatchNoverLmax 0.04 --alignIntronMin 20 --alignIntronMax 1000000 --alignMatesGapMax 1000000 --alignSJoverhangMin 8 --limitSjdbInsertNsj 2500000 --alignSJDBoverhangMin 1 --sjdbScore 1 --sjdbFileChrStartEnd hg19-150/sjdbList.out.tab --sjdbGTFfile gencode.v19.annotation.gtf --peOverlapNbasesMin 10 --alignEndsProtrude 10 ConcordantPair.” Raw count data for a total of 55,765 genes were obtained using htseq 0.11.4 using the default parameters and the option “--stranded=reverse.” Low-count genes were removed by requiring more than 15 reads in at least 2 samples for each gene across the 12 samples, and the remaining 15,612 genes were used for the subsequent analyses. Differentially expressed genes were identified on the basis of the criteria of adjusted *P* < 0.05 and shrunken log_2_ fold change (LFC) > log_2_(1.5) using DESeq2 (*65*). Shrunken LFC was obtained using the adaptive shrinkage estimator. Wald test was used to detect differentially expressed genes from the pairwise comparison between two contrasting groups. Fold inductions of differentially expressed genes are listed in table S4. The GEO accession number for the genomic data is GSE162617.

#### Antibodies

The following are the antibodies used in this study: NIPBL (Bethyl Laboratories, A301-779A; Thermo Fisher Scientific, MA1-72534), YY1 (Active Motif, 61779), H3K27ac (Active Motif, 39133), GR (Santa Cruz Biotechnology, sc-393232; Thermo Fisher Scientific, MA1-510), SMC1a (Bethyl Laboratories, A300-055A; Abcam, ab133643), SMC3 (Abcam, ab9263), RAD21 (Abcam, ab992), H2B (Abcam, ab61250), and tubulin (Abcam, ab6160).

## Notes

View/request a protocol for this paper from *Bio-protocol*.
