# Supplementary material for: The glucocorticoid receptor associates with the cohesin loader NIPBL to promote long-range gene regulation
Source: Sci Adv. 2022 Mar 30;8(13):eabj8360. doi: 10.1126/sciadv.abj8360 (PMC8967222; doi:10.1126/sciadv.abj8360)
Supplement: Supplementary file 1 — Figs. S1 to S7 Tables S1 to S6 [file sciadv.abj8360_sm.pdf]

Science Advances
AAAS

Supplementary Materials for

The glucocorticoid receptor associates with the cohesin loader NIPBL to promote long-range gene regulation

Lorenzo Rinaldi*, Gregory Fettweis, Sohyoung Kim, David A. Garcia, Saori Fujiwara, Thomas A. Johnson, Theophilus T. Tettey, Laurent Ozbun, Gianluca Pegoraro, Michele Puglia, Blagoy Blagoev, Arpita Upadhyaya, Diana A. Stavreva, Gordon L. Hager*

*Corresponding author. Email: hagerg@exchange.nih.gov (G.L.H.); lorenzo.rinaldi@nih.gov (L.R.)

Published 30 March 2022, *Sci. Adv.* **8**, eabj8360 (2022)
DOI: 10.1126/sciadv.abj8360

The PDF file includes:

Figs. S1 to S7
Tables S1 to S6
Legends for tables S1 to S6

Other Supplementary Material for this manuscript includes the following:

Tables S1 to S6

Figure S1: Cohesin binds chromatin in a hormone-dependent manner

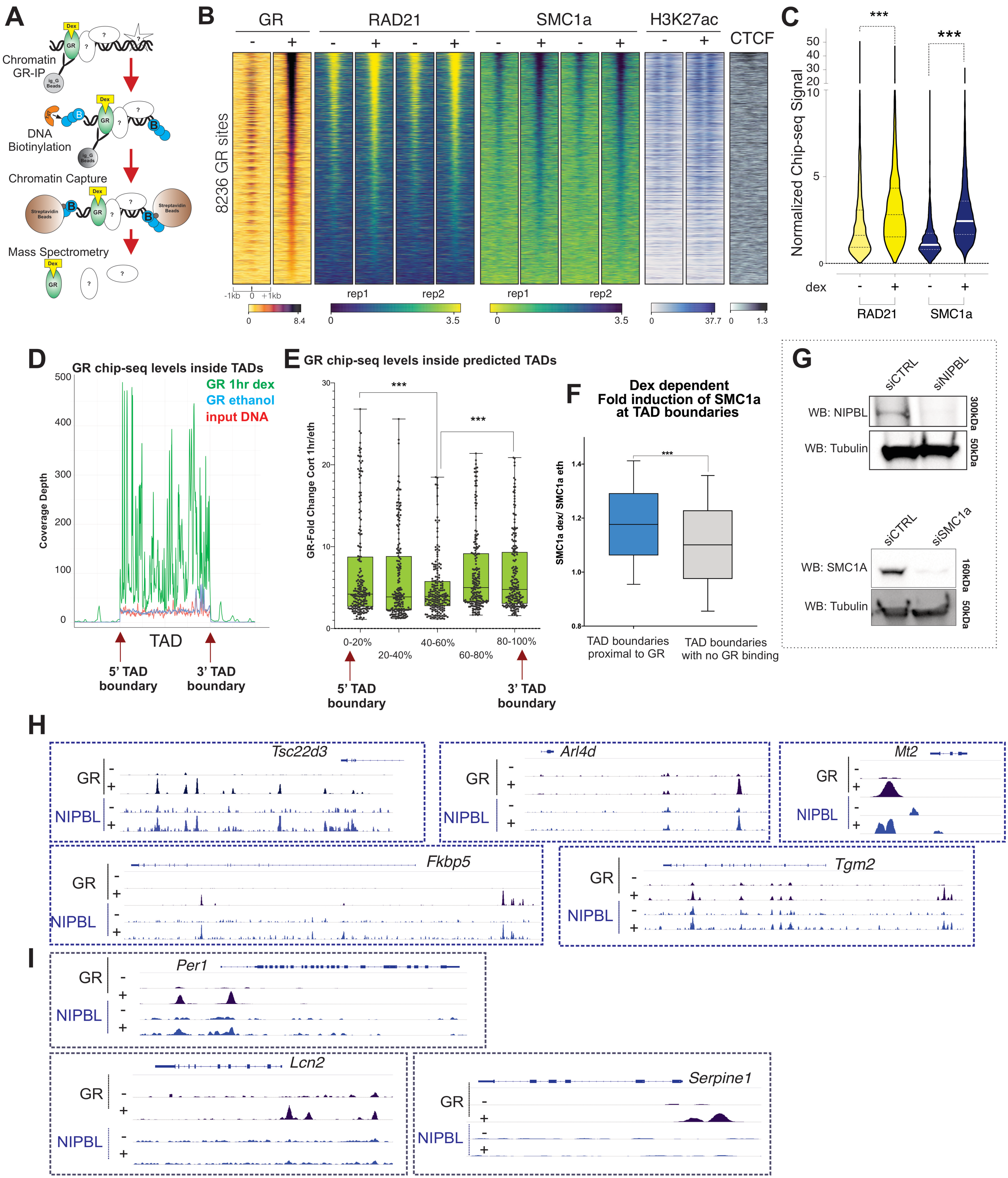

Figure S1, related to Figure1

(A) Schematic illustration of the GR ChIP-SICAP protocol. (B) ChIP-seq heatmaps of GR, RAD21, SMC1, H3K27ac and CTCF before and after 1hr of dexamethasone treatment. Normalized tag count was aligned over the 8236 GR chromatin location previously identified and published. Each dataset is normalized over 10million reads. (C) Tag quantification of RAD21 and SMC1a chip-seq before and after dexamethasone treatment at 8236 GR bound sites. Violin plots combine 2 replicates for each experiment. Data are normalized over 10million reads. (D) GR binds prevalently close to the CTCF TAD boundaries. GR chip-seq levels inside the predicted TADs domains identified by CTCF motif orientation analysis. Graph shows 3000 TADs that contain at least one GR peak. (E) Quartile analysis of the GR chip-seq levels inside the predicted TADs domains identified by CTCF motif orientation analysis (*31*). 0-20% is the first quartile of the TADs domain, 20-40% is the second quartile, 40-60% is the third quartile, 60-80% is the fourth quartile, 80-100% is the fifth and last TAD quartile. Pvalues derived using the Wilcoxon test. (F) Tag quantification of dexamethasone treatment induction of SMC1a at TAD boundaries that are close or not (within a 30kb window) to GR bound locations. p-value e2-26 derived using the Wilcoxon test. (G) Western blot assessing the knockdown efficiency of the siRNA treatment (72hrs) against NIPBL or SMC1A. (H-I) Genome browser screenshots of GR and NIPBL chip-seq data before and after dexamethasone treatment at the genes induced after GCs treatment (shown in figure 2G and figure 4). The genes in (H) are co-bound by both GR and NIPBL while the genes in (I) are not co-bound by NIPBL, suggesting these genes are activated by other mechanisms and possibly other GR co-factors.

Figure S2: Evidences of loop extrusion at GR-responsive enhancer elements

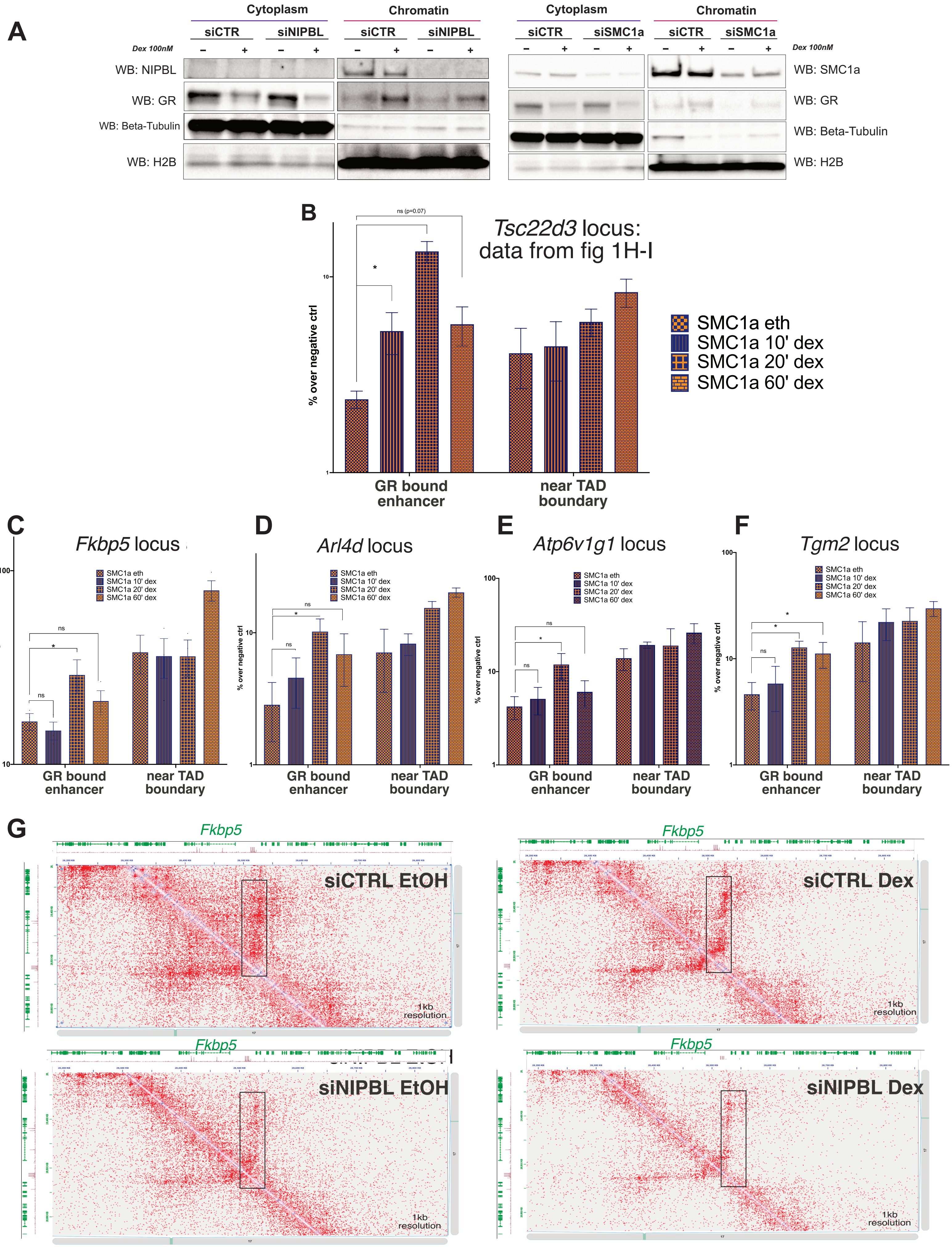

Figure S2, related to Figure1 and Figure4

(A) Cellular fractionation of cells depleted for NIPBL or SMC1a after one hour of dexamethasone treatment (100 nM). The purity of the cytoplasmic and chromatin fractions was assessed by blotting the beta-Tubulin and H2B protein, respectively. Knockdown of the NIPBL or SMC1A reduces the amount of GR bound to the chromatin fraction after dexamethasone treatment. Furthermore, dexamethasone treatment does not induce global changes in NIPBL or SMC1A chromatin binding, suggesting that their locus-specific enhanced chromatin binding is exclusive to the chromatin sites bound by GR. (B) ChIP qRT-PCR for SMC1a after ethanol, 10’, 20’ or 60’ of dexamethasone treatment (three biological replicates) at *Tsc22d3* GR-bound enhancer and the proximal TAD boundary. Same data of Figure 1H-I. (C-F) ChIP qRT-PCR for SMC1a after ethanol treatment, 10’, 20’ and 60’ of 10nM dexamethasone at different genomic loci with enhancers bound by GR and the proximal TAD boundaries near to GR-target genes *(Tsc22d3, Fkbp5,Arl4d, At6v1g1, Tgm2)*. Statistical tests are obtained running Mann-Whitney test on three biological replicates. (G) Micro-c supplementary data: Obversed/Expected contact frequencies of the Topological Associated domain that includes the GR-target gene *Fkbp5*. Dexamethasone treatment strongly potentiates the architectural stripes connecting GR-bound enhancers to the TAD boundary, supporting a role for GR and NIPBL to mediate DNA extrusion. All datasets represent two independent biological replicates and are normalized to the same intensity using Juicebox at 1000bp resolution.

Figure S3: NIPBL loss alter GR-bound chromatin interactions and gene regulation:

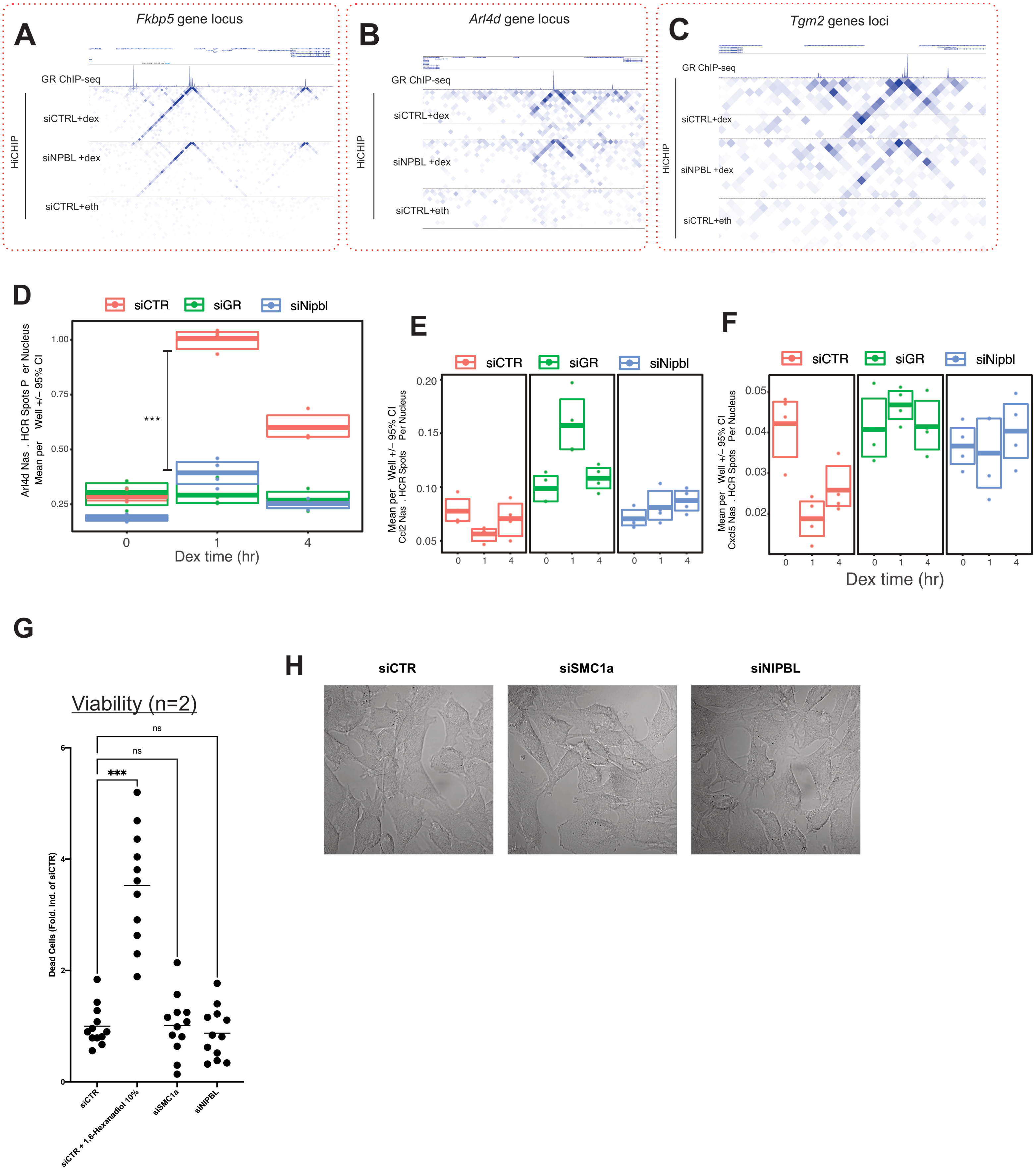

Figure S3, related to Figure2 and Figure4

(A,B,C) WashU Genome Browser screenshots of HiC transformed maps (using HiC-Pro) from the GR-HiChIP interactomes. Screenshots of the gene locus related to the GR-target genes: *Tsc22d3, Fkbp5, Arl4d and Tgm2.* Hi-C data are normalized, and blue squares represent the 5kb resolution. (D) Nascent RNA of ARL4D, a GR activated gene using the Hybridization chain reaction as explained in detail in Figure 3. The graph shows the average number of active nascent RNA foci por well at 0,1,4 hr of dexamethasone treatment for ARL4D after 72hrs of siRNAs siCTRL , siGR and siNIPBL cells. Pvalue<0.00001 derived from two-way Anova test both for siGR and siNIPBL cells. (E-F) Average number of active nascent RNA foci por well before and after dexamethasone treatment for two GR repressed pro-inflammatory genes after 72hrs of siCTRL, siGR and siNIPBL cells. CCL2 (E) and CXCL5 (F). CCL2 pvalue is 0.06 and CXCL5 is <0.05. Both derived from two-way Anova test both for siGR and siNIPBL cells. (G) Cells were plated 72 hours after the indicated siRNA treatment. The positive control has been designed by incubating the cells for 1 hour with 1,6 hexanadiol 10%. (H) Differential Interference Contrast microscopy (DIC) was performed to investigate the morphology of samples treated with the indicated siRNAs on a LSM-780 microscope (Zeiss)

Figure S4, Nuclear Localization of Transcribing Foci

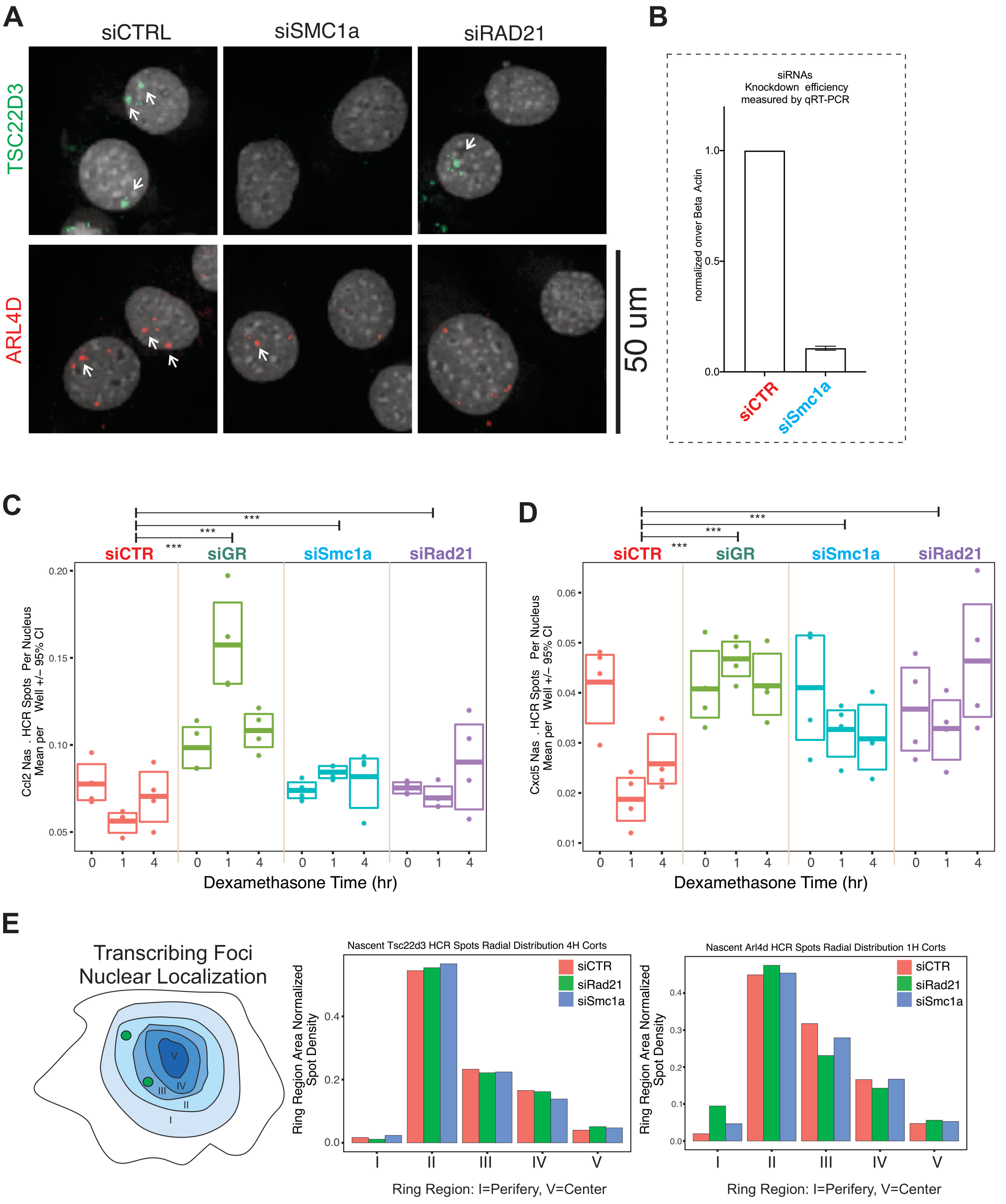

Figure S4, related to Figure4

(A) Representative images of transcribing foci of the nascent RNA of TSC22d3 (green channel) and ARL4D (red channel) in siCTRL, siSMC1a, siRAD21 cells. Scale bar is 50um. (B) qRT-PCR assessing the knockdown efficiency of siRNA treatment against SMC1a in three biological replicates. Ct values are normalized over beta-actin, used as a loading control. (C,D) Average number of active nascent RNA foci por well before and after dex treatment for two GR repressed pro-inflammatory genes after 72hrs of siRNA against no target, GR and NIPBL. CCL2 (C) and CXCL5 (D). CCL2 Pvalue<0.00001 for siSMC1a and siRAD21. CXCL5 Pvalue<0.001 for siSMC1a and siRAD21. All Pvalues are derived from two-way Anova test. (E) Illustration of the analysis of the transcribing foci localization respect to the nuclear membrane: nuclei are divided in five circular areas, I closer to the nuclear membrane and V at the nuclei center. Custom-built analysis quantified areas within which each transcribing location was spotted, for each specific cell. (E) Most transcribing loci are found proximal to the nuclear membrane, while this localization is unaffected by the loss of SMC1a or RAD21. No statistically significant difference was found between samples.

Figure S5

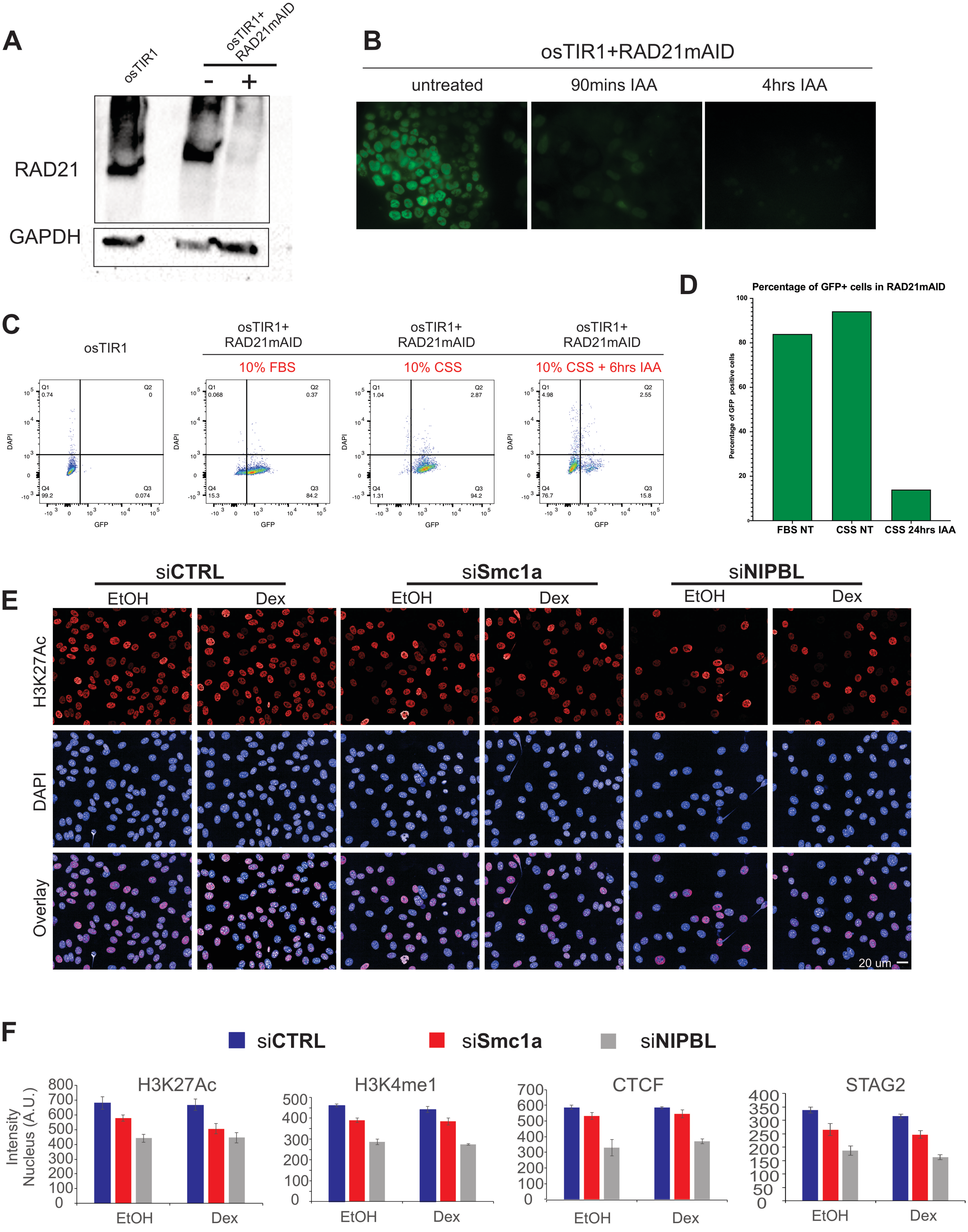

A
osTIR1
osTIR1+ RAD21mAID
- +
RAD21
GAPDH
B
osTIR1+RAD21mAID
untreated
90mins IAA
4hrs IAA
C
osTIR1
osTIR1+ RAD21mAID
10% FBS
10% CSS
10% CSS + 6hrs IAA
DAPI
GFP
D
Percentage of GFP+ cells in RAD21mAID
Percentage of GFP positive cells
FBS NT
CSS NT
CSS 24hrs IAA
E
siCTRL
siSmc1a
siNIPBL
EtOH
Dex
H3K27Ac
DAPI
Overlay
20 um
F
siCTRL
siSmc1a
siNIPBL
Intensity Nucleus (A.U.)
H3K27Ac
H3K4me1
CTCF
STAG2
EtOH
Dex

Figure S5, related to Figure 5

(A) Western blot for RAD21 in HCT116 osTIR1 cells and HCT116 osTIR1+RAD21mAID-clover (-/+ 2hrs of auxin). GAPDH was used as a loading control. (B) Representative images of HCT116 osTIR1+RAD21mAID-clover fluorescence after 0, 90mins and 240mins of IAA treatment. (C) Flow cytometry of HCT116 osTIR1+RAD21mAID-clover before and after IAA treatment. (D) Cells grown in Charcoal stripped FBS show an enhanced expression of the tagged RAD21-clover protein compared to FBS grown cells, most likely for auxin contamination (or similar activating hormones) in normal FBS. All our experiments using HCT116 osTIR1+RAD21mAID-clover are done using McCoy medium supplemented with 10% Charcoal stripped FBS. (E) Representative immunofluorescence images of H3k27ac - red channel- (1hr ethanol or dexamethasone treated) of 3134 cells after 5 days of siRNA transfection against non-target sequence (siCTRL), SMC1a, RAD21. Nuclei are marked in blue by DAPI (4′,6-diamidino-2-phenylindole). (F) Immunofluorescence nuclear quantification staining done for H3K27ac, H3K4me1, CTCF and STAG2 in 3134 cells after 5 days of siRNA transfection against non-target sequence (siCTRL), SMC1a, RAD21. Quantifications are normalized over the number of total cells.

FigureS6

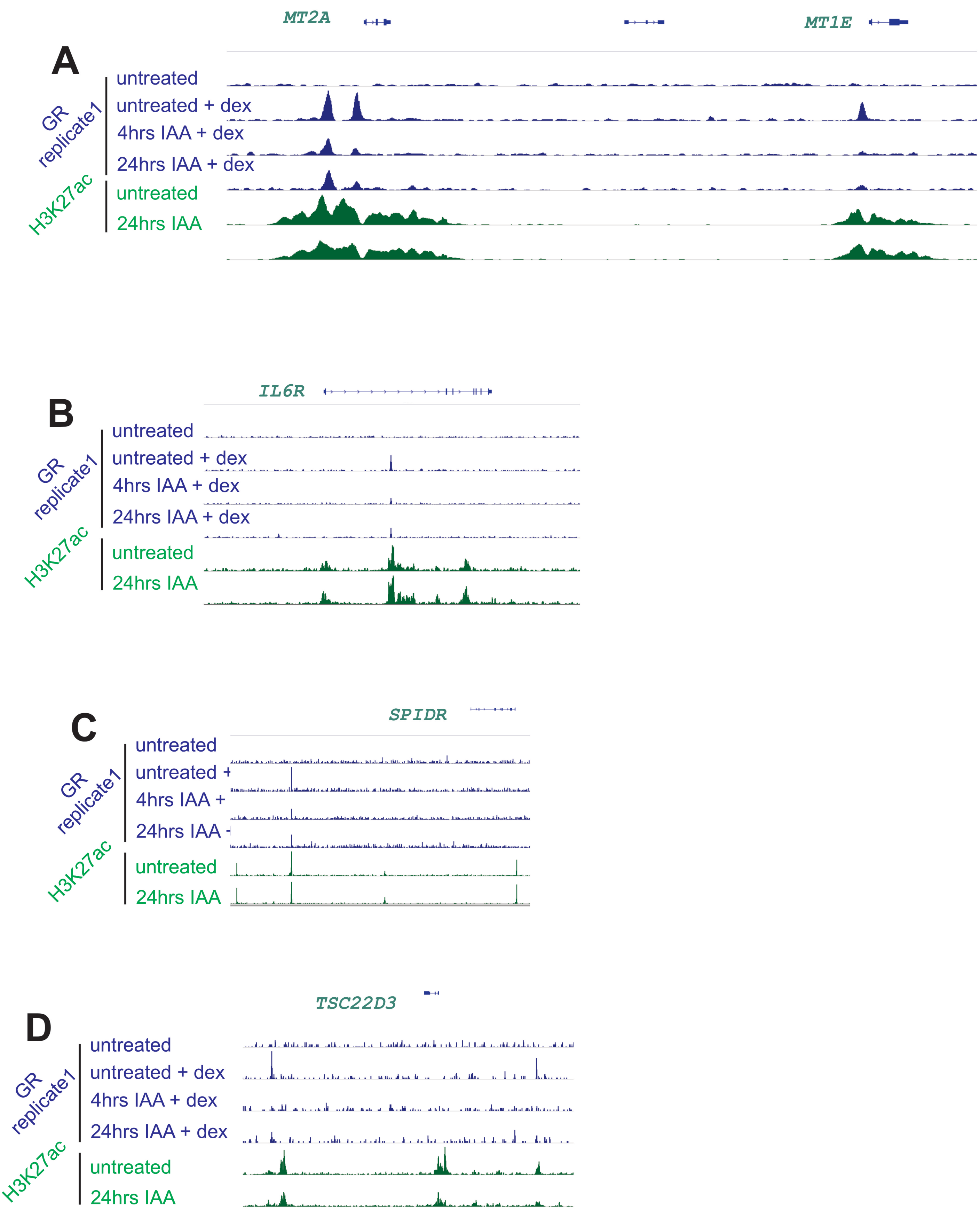
A
MT2A
MT1E
GR replicate1
untreated
untreated + dex
4hrs IAA + dex
24hrs IAA + dex
H3K27ac
untreated
24hrs IAA
B
IL6R
GR replicate1
untreated
untreated + dex
4hrs IAA + dex
24hrs IAA + dex
H3K27ac
untreated
24hrs IAA
C
SPIDR
GR replicate1
untreated
untreated +
4hrs IAA +
24hrs IAA
H3K27ac
untreated
24hrs IAA
D
TSC22D3
GR replicate1
untreated
untreated + dex
4hrs IAA + dex
24hrs IAA + dex
H3K27ac
untreated
24hrs IAA

Figure S6, related to Figure 5

(A-D) Genome browser screenshots of chromatin locations bound by GR in HCT116 RAD21mAID cells before and after auxin treatment. Two biological replicates are combined for each sample. Data are normalized at 10 million reads and plotted at the same scale.

Figure S7

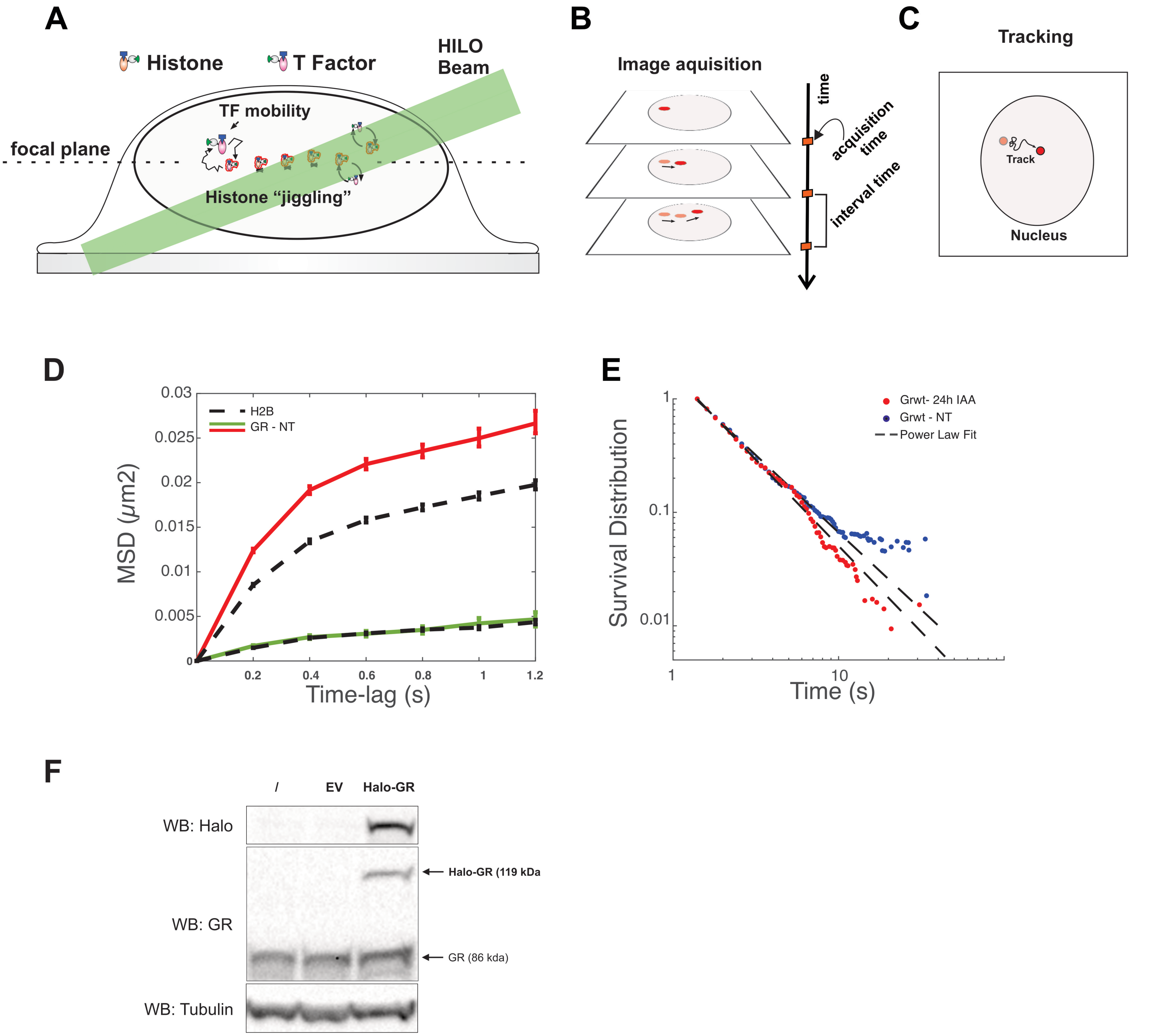

A
Histone
T Factor
HILO
Beam
TF mobility
focal plane
Histone "jiggling"
B
Image aquisition
time
acquisition
time
interval time
C
Tracking
Track
Nucleus
D
H2B
GR - NT
MSD (μm2)
0.03
0.025
0.02
0.015
0.01
0.005
0
0.2
0.4
0.6
0.8
1
1.2
Time-lag (s)
E
Grwt- 24h IAA
Grwt - NT
Power Law Fit
Survival Distribution
1
0.1
0.01
1
10
Time (s)
F
/
EV
Halo-GR
WB: Halo
WB: GR
WB: Tubulin
Halo-GR (119 kDa
GR (86 kda)

Figure S7, related to Figure 7

(A) In a representative HiLo microscopy setup, a laser illuminates the cell at an angle which decreases out of focus fluorescence compared to widefield microscopy. Tagged proteins in the laser light sheet are excited and particles in the focal plane can be tracked. Histones will be mostly immobile "jiggling" in the nucleus while TFs will present different modes of diffusion and interactions with chromatin. (B) A snapshot of the nucleus is taken every interval time until molecules are photobleached. Each snapshot is taken with a particular acquisition time and the TF trajectory is reconstructed. (C) Schematic illustration of the tracking process for single molecule TF into the nuclear space. (D) MSD versus lag time for chromatin binding (Green) and Confinement (Red) states exhibited by GR-Halo conjugated with JF_{549} and treated with 100 nM Dex (15-120 minutes prior to imaging) and H2B-Halo. The chromatin bound states present similar MSD tracks as expected from the biology. (E) Dwell time distribution for the confinement population of GR-Halo treated with 100nM dex, before (Blue dots) and after 24hrs of auxin treatment (Red). The distribution is fitted to a power-law model (dashed lines). (E) MSD versus lag time for chromatin binding (Green) and confinement (Red) of GR-Halo conjugated with JF_{549} and treated with 100 nM Dex before (Solid lines) and after 24hrs of auxin treatment (Dashed lines). MSDs are calculated from 7-frame track segments, with 200 ms acquisition interval, 10 ms exposure. (F) Western blot showing the level of expression of the exogenous tagged-hGR transfected into the HCT116 cells containing mAID-tagged RAD21. The hGR expression is comparable of the endogenous hGR. The plasmid carrying Halo-hGR has been engineered to keep the expression of Halo-hGR as low as possible by containing a CER region and a CMVd1 promoter. Beta-Tubulin is used as loading control.

Supplementary Tables

Supplementary Table 1

Significant GR interactors at chromatin level, identified by ChIP-SICAP, are listed in Supplementary Table 1

Supplementary Table 2

Genomics results of Cohesin after dexamethasone treatment are listed in Supplementary Table 2

Supplementary Table 3

GR-HiChIP significant interaction at q value 0.01 for samples ethanol, siCTRL and siNIPBL are listed in Supplementary Table 3.

Supplementary Table 4

TNF-alpha RNA-seq identified differential expressed genes are listed in Supplementary Table 4.

Supplementary Table 5

Differential chromatin interactions identified by Micro-C between siCTRL EtOH and siCTRL Dex.

Supplementary Table 6

Architectural stripes found by micro-C using Stripenn in the four samples. GR-bound architectural stripes. Listed in supplementary table 6
